# A multi-country, prospective cohort study to measure rate and risk of relapse among children recovered from severe acute malnutrition in Mali, Somalia, and South Sudan: a study protocol

**DOI:** 10.1186/s40795-022-00576-x

**Published:** 2022-08-24

**Authors:** Sarah King, Lauren D’Mello-Guyett, Ellyn Yakowenko, Bram Riems, Karin Gallandat, Sherifath Mama Chabi, Feysal Abdisalan Mohamud, Khamisa Ayoub, Ahmed Hersi Olad, Bagayogo Aliou, Anastasia Marshak, Indi Trehan, Oliver Cumming, Heather Stobaugh

**Affiliations:** 1 Action Against Hunger, New York, NY USA; 2 Environmental Health Group, Department for Disease Control, Faculty of Infectious and Tropical Diseases, London School of Hygiene and Tropical Medicine, London, UK; 3 Action Contre La Faim, Paris, France; 4 Acción Contra El Hambre, Madrid, Spain; 5 Nutrition Department, Ministry of Health for the Republic of South Sudan, Juba, South Sudan; 6 Research Department, Federal Ministry of Health for the Federal Republic of Somalia, Mogadishu, Somalia; 7 Nutrition Sub-Directorate, General Directorate of Health and Public Hygiene, Ministry of Health and Social Development for the Republic of Mali, Bamako, Mali; 8 Feinstein International Center, Tufts University, Boston, MA USA; 9 Departments of Pediatrics, Global Health, and Epidemiology, University of Washington, Seattle, WA USA; 10 Tufts University, Boston, MA USA

**Keywords:** Severe acute malnutrition, Wasting, Kwashiorkor, Marasmus, Community-based management of acute malnutrition, Relapse, Post-discharge outcome, Water, sanitation, and hygiene, Enteric infection

## Abstract

**Background:**

The Community-Based Management of Acute Malnutrition (CMAM) model transformed the treatment of severe acute malnutrition (SAM) by shifting treatment from inpatient facilities to the community. Evidence shows that while CMAM programs are effective in the initial recovery from SAM, recovery is not sustained for some children requiring them to receive treatment repeatedly. This indicates a potential gap in the model, yet little evidence is available on the incidence of relapse, the determinants of the phenomena, or its financial implications on program delivery.

**Methods:**

This study is a multi-country prospective cohort study following “post-SAM” children (defined as children following anthropometric recovery from SAM through treatment in CMAM) and matched community controls (defined as children not previously experiencing acute malnutrition (AM)) monthly for six months. The aim is to assess the burden and determinants of relapse to SAM. This study design enables the quantification of relapse among post-SAM children, but also to determine the relative risk for, and excess burden of, AM between post-SAM children and their matched community controls. Individual -, household-, and community-level information will be analyzed to identify potential risk-factors for relapse, with a focus on associations between water, sanitation, and hygiene (WASH) related exposures, and post-discharge outcomes. The study combines a microbiological assessment of post-SAM children’s drinking water, food, stool via rectal swabs, dried blood spots (DBS), and assess for indicators of enteric pathogens and immune function, to explore different exposures and potential associations with treatment and post-treatment outcomes.

**Discussion:**

This study is the first of its kind to systematically track children after recovery from SAM in CMAM programs using uniform methods across multiple countries. The design allows the use of results to: 1) facilitate understandings of the burden of relapse; 2) identify risk factors for relapse and 3) elucidate financial costs associated with relapse in CMAM programs. This protocol’s publication aims to support similar studies and evaluations of CMAM programs and provides opportunities for comparability of an evidence-based set of indicators for relapse to SAM.

**Supplementary Information:**

The online version contains supplementary material available at 10.1186/s40795-022-00576-x.

## Background

Acute malnutrition (AM) remains a major global health problem affecting an estimated 45.4 million children under 5 years of age [1]. Of these, some 31.8 million have moderate acute malnutrition (MAM) and 13.6 million have severe acute malnutrition (SAM) [1], and the true burden of the disease is likely much higher due to weak surveillance. Children suffering from SAM are at high risk of near-term death, approximately 12 times higher than non-malnourished children [2]. Those with multiple forms of malnutrition, such as concurrent wasting, and stunting, are at an even higher risk of near-term death [3].

The introduction of the Community-Based Management of Acute Malnutrition (CMAM) model over the past two decades has significantly improved standard practice for the treatment of AM [4]. SAM cases without medical complications are now predominantly managed closer to the community, in outpatient therapeutic programs (OTP) and MAM cases in supplementary feeding programs (SFP). This innovation has increased coverage, improved treatment outcomes, and reduced costs [5–7]. While progress has been made, most programs focus on initial recovery only, without addressing post-discharge outcomes. There is growing evidence of poor post-discharge outcomes including high rates of relapse and mortality, after initial SAM recovery [8, 9]. This calls into question the effectiveness and efficiency of the approach if CMAM programs are re-treating the same children repeatedly.

Few studies have followed children longitudinally after recovery from SAM and discharge from CMAM programs causing reliable rates for post-discharge outcomes to be limited [8, 9]. Among the few studies that have been conducted, inconsistent definitions of relapse and varying methodologies for measuring post-discharge outcomes have led to a small, incoherent evidence base. A recent literature review on relapse following SAM recovery demonstrates the wide range of reported relapse rates globally: 0–37% [8]. Nonetheless, these studies do indicate that a large portion of children likely maintain a high degree of vulnerability after recovery from SAM, including heightened mortality and morbidity risk, leaving post-SAM children particularly susceptible to poor post-discharge outcomes [10–14].

One potentially important determinant of SAM relapse following discharge from CMAM programs are the environmental conditions in the homes and communities and enteric infections. Unsafe drinking water, sanitation, and hygiene (WASH) at the household level exposes children to various infectious disease risks which in turn threaten continued nutrition and health outcomes among discharged children. One study in Niger, found that the length of stay in CMAM programs was much longer among children in households with inadequate drinking water access [15]. Previous studies have shown that treating household-level drinking water and/or promoting caregiver hygiene improves outcomes during SAM treatment [16, 17]. Additionally, one study found sustained recovery following MAM treatment was associated with improved household-level WASH conditions [18]. These findings suggest that interventions to improve household WASH conditions may help to reduce the risk of SAM relapse following discharge but to date there have been no rigorous studies which link domestic environmental conditions to risk of relapse directly.

There is a clear need to better understand post-discharge outcomes following treatment under the CMAM model and to identify potential improvements that may make these programs more effective and efficient in achieving sustained recovery. For example, a reduction in relapse rates may free resources to increase coverage for new admissions to receive life-saving treatment. Identifying the burden of relapse and the resources currently being allocated to re-treating the same children for multiple episodes of SAM over a relatively short period of time is a necessary first step in improving current programming to reduce relapse rates.

The aim of this research is to assess the burden and determinants of SAM relapse across three settings with a high prevalence of AM. The specific study objectives are to: 1) compare the cumulative incidence of SAM among children after recovery from SAM in outpatient treatment programs with the cumulative incidence of SAM among children who did not previously experience acute malnutrition; 2) identify child- and household-level factors associated with SAM relapse; 3) assess the financial costs of re-treating children who experience relapse to SAM within six months of recovery from SAM through CMAM programs; and 4) infer possible CMAM programmatic factors that may influence SAM relapse when comparing relapse rates across the three different programs and contexts. A nested sub-study will focus on the role of WASH in relapse to SAM, and has three specific objectives, to: 1) assess the association between household and community level WASH conditions and risk of relapse; 2) assess the burden of enteric pathogen detection at discharge from CMAM programs and its association with subsequent relapse; and 3) assess bacterial contamination of food and drinking water consumed by children post discharge and its association with subsequent relapse.

## Methods

### Study design

This study is a multi-country prospective cohort study following “post-SAM” children (defined as children following anthropometric recovery from SAM) and matched community controls (defined as children who did not previously experience AM) monthly for six months. During the follow-up period, children are assessed for AM as well as linear growth, morbidity, and mortality outcomes. Follow-up procedures and data collection are identical across both post-SAM and control groups. This study design enables the quantification of relapse among post-SAM children, but also to determine the relative risk for, and excess burden of, AM between post-SAM children and their matched community controls.

Individual-, household-, and community-level information will be analyzed to identify potential risk-factors for relapse, with a specific focus on associations between WASH-related exposures and post-discharge outcomes. The study will combine a microbiological assessment of post-SAM children’s drinking water, food, enteric infections, immune function, and antibiotic resistance to explore different exposures and their potential associations with treatment and post-treatment outcomes.

### Study setting

This study is being implemented in three countries: Mali, Somalia and South Sudan. Each represents a context with concurrent and reoccurring humanitarian crises, frequent ‘hunger gap’ seasons, and a high prevalence of AM [19]. Locations also represent a variety of climates, livelihoods, cultures, and political settings.

#### Kayes, Mali

The district of Kayes is positioned in southwest Mali. The region is relatively stable, but health services are frequently disrupted by ongoing strikes at both national and regional levels. Communities also see significant flooding during the region’s rainy season, cutting them off from essential services. The rainy season is accompanied by a dry cool season and a dry hot season [20]. The recent IPC Acute Malnutrition analysis, covering June 2021 – August 2022, showed the district to be in a serious nutritional situation [21]. A 2020 survey in Kayes shows the global acute malnutrition (GAM) prevalence at 5.6%, with 0.3% consisting of SAM [22]. A total of 50 health centers are located throughout the district and run by the Ministry of Health. Of these facilities, nine clinics are included in this study.

#### Mogadishu, Somalia

Located in the Banadir Region, Mogadishu is home to approximately 500,000 internally displaced persons. The area consistently experiences high rates of acute malnutrition due to significant food insecurity and ongoing instability. Banadir typically experiences four seasons; a hot dry season, a main rainy season, a cool dry season and a second rainy season, but the seasonal rains frequently fail resulting in widespread severe drought [23]. The 2020 SMART survey found the GAM prevalence to be at 9%, with 1.3% identified as SAM [22]. As of the most recent Integrated Food Security Phase Classification (IPC) Analysis from April 2022, the Banadir Region is classified as Phase 4 (Emergency) [24]. The nutrition site in Khada, one of two Action Against Hunger supported SAM outpatient treatment program sites in Mogadishu, is included in this study.

#### Aweil East, South Sudan

Aweil East County is situated in the state of Northern Bahr el Ghazal. The region sees one primary rainy season with a short cool dry season and a hot dry season, leading communities to migrate between the highlands and lowlands depending on the season to access water [25]. As of a 2021 SMART survey, the GAM and SAM prevalence was 13.1% and 2.6% respectively [26]. The county is routinely classified as IPC Phase 3 (Crisis) or IPC Phase 4 (Emergency), signifying widespread food insecurity and high rates of acute malnutrition. The region was classified as Phase 4 in 2022 [27]. Within Aweil East, Action Against Hunger runs a total of 13 nutrition sites that are paired with existing Ministry of Health run health facilities. A total of six nutrition sites are included in the study.

### Period of recruitment

Recruitment of study participants began in April of 2021 and enrollment is expected to be completed in July of 2022. All data collection is expected to be completed by January of 2023, at which point data will be analyzed.

### Study population

Children aged 6–47 months at the point of recovery from SAM and discharge from an OTP or SFP within integrated management of acute malnutrition (IMAM) services or a CMAM program are being enrolled into the study. Following WHO recommendations at the start of this study [28], recovery from SAM is defined as MUAC ≥ 125 mm, WHZ ≥ -2, and/or no edema for two consecutive weeks. In this study, post-discharge, post-recovery, and post-SAM are defined identically as the period following discharge as recovered from SAM. A control group of children who did not previously experience AM matched by age, sex, location, and timing of enrollment as their post-SAM counterparts are also being enrolled. The following inclusion and exclusion criteria are in use.

For post -SAM children, the inclusion criteria are:

- Age 6–47 months
- Being discharged from treatment as recovered according to the global WHO definition of recovery from SAM: with a MUAC ≥ 125 mm, WHZ ≥ -2 and/or no edema for two consecutive weeks
- Caregiver planning to stay in the area and willingness to attend the clinic for the subsequent 6 months for follow-up visits
- Caregiver having signed an informed consent form for his/her child
  For non-acutely malnourished community control children, the inclusion criteria are:
- Matched to a post-SAM child by age (within ± 3 months between ages 6–11 months and ± 6 months for children ages 12–47 months), gender, location, and timing of enrollment into the study
- Matched to a post-SAM child by community of residence, such that they live in the same general location (surrounding communities) of the clinic site where the matched post-SAM child attended for treatment
- MUAC ≥ 125 mm, WHZ ≥ -2, and no edema
- No history of an identified episode of acute malnutrition in the past 6 months
- Caregiver planning to stay in the area and willingness to attend the clinic for the subsequent 6 months for follow-up visits
- Caregiver having signed an informed consent form for his/her child
  For post-SAM children, the exclusion criteria are:
- Presence of a chronic or congenital disease (not including HIV infection or TB) or disability that affects growth, ability to consume food, or anthropometric measurements (e.g., cerebral palsy)
- Initial OTP admission was preceded by enrollment in an inpatient, hospital, or stabilization center where the child was being treated for SAM with medical complications.
  For non-acutely malnourished community control children, the exclusion criteria are:
- Presence of a chronic or congenital disease (not including HIV infection or TB) or disability that affects growth, ability to consume food, or anthropometric measurements (e.g., cerebral palsy)

In the Mali location, the national protocol defines recovery from SAM as MUAC ≥ 125 mm, WHZ ≥ -1.5 and/or no edema. Therefore, in Mali, when children are treated for SAM according to WHZ, they are discharged as recovered at WHZ ≥ -1.5, not WHZ ≥ -2. The final analyses will explore if and how this sub-group of children will have different results.

### Data collection procedures

#### Main study

Recruitment of the post-SAM cohort occurs at OTP or SFP sites supported by Action Against Hunger. Recruitment of the control children occurs through referrals from trained community health workers (CHWs) performing regular nutrition screening in the communities. Each week, these CHWs provide a list of age ranges and genders that match the post-SAM children who were enrolled in the study the previous week. The CHWs then use these age and gender parameters to guide the recruitment of matched controls. The timing of control children enrollment matches as closely as possible to the timing of matched post-SAM children enrollment, which is occurring on a rolling basis.

Once enrollment criteria are confirmed and informed consent has been taken, trained data collectors evaluate the child’s acute malnutrition status by measuring anthropometry and assessing edema. Standard methodologies for anthropometric measurements are used: weight is measured in duplicate using an electronic scale to the nearest 0.01 kg; length is measured in duplicate to the nearest 0.1 cm using a rigid length board; and MUAC is measured in duplicate with a standard insertion tape to the nearest 0.1 cm. Median values across replicate measures will be used for final analysis. Participants are evaluated for edematous malnutrition (kwashiorkor) by assessing for bilateral pitting edema.

A predefined and pre-tested structured enrollment questionnaire is administered in the local language to collect variables related to the child and caregiver. Information collected includes child feeding practices, child health history, childcare practices, household demographic information, household WASH, and household food security using the Household Hunger Scale [29]. For post-SAM children, medical records during initial SAM treatment prior to enrollment are obtained to collect all information regarding each child’s health and nutrition parameters throughout SAM treatment. This includes weekly anthropometrics measurements (weight, height, MUAC, and edema), length of treatment, and symptoms of co-morbidities present during treatment.

All caregivers, including both post-SAM and control groups, are asked to bring children back to the nutrition clinic site for a monthly follow-up visits post-discharge for a period of six months. Data collection schedules and procedures are identical across both study groups. At each follow-up visit, the child’s anthropometric measurements (weight, length, and MUAC) are collected and edema reassessed. An interview with the caregiver is conducted to collect information regarding child illness symptoms, receipt of any medical care, participation in any other assistance programs, household food security, household WASH, etc.

At each follow-up point, the child is classified with one of the following outcomes:

•Without acute malnutrition, defined as having a MUAC ≥ 125 mm, WHZ ≥ -2, and without edema
•Relapsed to SAM, defined as having a MUAC < 115 mm, WHZ < -3, and/or edema
•Relapsed to MAM, defined as having a MUAC of 115-124 mm or WHZ > -3 and ≤ -2 without edema
•Having died
•Defaulted, defined as failure to complete the follow-up visit within 3 weeks of the scheduled visit

If a child is identified to have either SAM or MAM, he/she is referred and treated accordingly. Clinical treatment data for all relapses is retrieved from the corresponding clinic. Regardless of whether the child relapses, the child will remain in the study for the full 6-month follow-up period.

Caregivers who miss scheduled follow-up visits are sought by CHWs at their homes and encouraged to return on the next day to complete the child’s scheduled follow-up visit. After two home visits by a CHW that fails to result in a completed follow-up visit by the participant, members from the study team travel to the home to either collect the data directly at the home or confirm that the study participant has defaulted for that scheduled follow-up visit. Therefore, default is defined as failing to complete a scheduled follow-up visit within three weeks of the originally scheduled visit. Lost to follow-up is defined as defaulting from a scheduled follow-up visit and failing to complete any further data collection.

All survey questions have been translated into the primary local language at each study site and back translated to confirm wording. All tools (Additional File 1) are verbally administered by trained field workers to the mother or primary caregiver of the post-SAM or control child. Survey data is collected on tablets using Open Data Kit (ODK), or on paper-based data collection tools designed specifically for the study. Paper-based data collection is entered to Microsoft Access weekly by research coordinators. A summary of the data collection procedures is outlined by cohort, location, and timing in Table 1.

**Table 1:** Schedule of activities for subjects by study group and location

	**Cohort**		**Location**			**Time**								
	**Post-SAM**	**Control**	**South Sudan**	**Mali**	**Somalia**	**SAM Treatment**			**Months Post-Discharge**					
						**Admission**	**Mid-Point**	**Recovery /** **Discharge**	**1**	**2**	**3**	**4**	**5**	**6**
**Clinic Sites**														
Enrollment Criteria Confirmed	X	X	X	X	X			X						
Informed Consent	X	X	X	X	X			X						
Enrollment Questionnaire	X	X	X	X	X			X						
Post-Discharge Follow-up Visit Questionnaire	X	X	X	X	X				X	X	X	X	X	X
Post-Discharge Follow-up Visit Anthropometric Measurements	X	X	X	X	X			X	X	X	X	X	X	X
Initial SAM Treatment Data (secondary data collection)	X		X	X	X			X						
Relapse AM Treatment Data (secondary data collection)	X	X	X	X	X				*(As needed)*					
Stool sample by rectal swab	X		X					X						
Dried Blood Spots (DBS)	X	X	X			X	X	X	X			X		
**Home Visit**														
Household WASH Questionnaire	X		X	X	X			X			X			X
Household Water Sample	X		X	X	X			X			X			X
Child Food Sample	X		X					X						
Structured observations of food preparation and feeding	X		X					X	X	X	X	X	X	X

### Costs sub-study

To gather data regarding the financial costs of relapse, financial data regarding the CMAM programmatic costs will be collected through a review of program financial records and interviews with relevant staff. All estimates will be confirmed with receipts, invoices, and other financial records when possible. Details of collecting and analyzing costing data will be detailed in a separate publication. Ultimately, this process will lead to the development of cost-efficiency results that will provide information related to the costs of re-treating children for relapse as an indication for the cost-efficiency of current CMAM protocols in achieving sustained recovery.

### WASH sub-study

The WASH sub-study involves additional data collection from the post-SAM study participants only (Table 2). In all three sites (Mali, Somalia and South Sudan), data collection includes: (1) a household WASH questionnaire and (2) collection and analysis of household drinking water samples, at three time-points across the follow-up period (enrollment, 3-months, and 6-months post-discharge). In only one site (South Sudan), the study collects: (1) child food samples at the household at time of SAM recovery from the CMAM program; (2) stool samples by rectal swab at time of SAM recovery from the CMAM program; and (3) dried blood spots (DBS) at the time of admission into OTP, four weeks into treatment, at the time of SAM recovery from the CMAM program, and at month one and month four post-discharge. Additionally, in South Sudan, to elucidate the food and food hygiene related risks among this study population, we are conducting (4) structured observations of child food preparation and feeding throughout the post-discharge period.

**Table 2:** Location, Sample Size, and Timing of WASH Sub-study Data Collection

	**Dried blood spots (DBS)**	**Stool sample via rectal swab**	**Household WASH visit and water sample**	**Food sample**	**Structured observations of food preparation**
**Time of data collection**	During treatment, enrollment, 1-, and 4-months post-discharge	Enrollment	Enrollment, 3-months post-discharge, 6-months post-discharge	Throughout post-discharge^a^	Throughout post-discharge
**Mali**	0	0	250	0	0
**Somalia**	0	0	250	0	0
**South Sudan**	300	614	614	614	200

^a^ Food samples will be collected from 614 HHs throughout data collection either during the enrollment household visit or during the structured food observations. There are also 200 randomly selected HHs that will also participate in the structured food observations. For these 200 HHs, the food sample will be collected at the end of the structured observation, which occurs at various points through the post-discharge period, as opposed to at enrollment. For the remaining 414 HHs, the food sample will be collected at the enrollment visit, if available

Laboratory analysis of environmental and clinical samples includes: quantification of fecal indicator bacteria (*E. coli*) in food and water samples; and detection of over 30 pathogens in food and stool samples using a customized real-time PCR assay (TaqMan Array Card). Exploratory laboratory analysis will also include describing the presence of clinically relevant antimicrobial resistance genes (ARGs) in food and stool samples on a small sub-set of samples; and, measurement of immune response to approximately five enteric pathogens and immune function using a bead-based multiplex assay in DBS samples. Exposure definitions for the WASH sub-study are listed in Supplementary Table 1.

### Primary study outcomes

The primary outcome of interest for this study is cumulative incidence of SAM, MAM, and AM (i.e., relapse incidence) over a six-month follow-up period among both the post-SAM and control groups. The secondary outcomes of interest will include incidence rate of SAM, MAM, and AM, and point prevalence of SAM, MAM, and AM throughout the six-month follow-up period across the post-SAM and control groups. Tertiary outcomes of interest for post-SAM and control groups will be time receiving treatment for SAM time until relapse; cumulative incidence and prevalence of morbidity; and changes in anthropometric measurements (e.g., WHZ, MUAC) throughout the six-month follow-up period. Primary and secondary outcome and exposure definitions are listed in Supplementary Tables 1, 2, 3.

### Sample size calculations

To identify the relative risk (RR) of post-SAM children becoming AM following SAM treatment (i.e., relapse incidence) in comparison with the general population for becoming AM (i.e., regular acute malnutrition incidence), a sample size was calculated to capture a statistical difference in the cumulative incidence of SAM between the post-SAM group and the community control group. Cumulative incidence of relapse to SAM in the post-SAM group was estimated to be approximately 12% while the cumulative incidence of SAM in the community control group will be 5% based on previous studies in in Ethiopia [30] and the Democratic Republic of the Congo [31]. The study is designed to take significant measures to limit loss to follow-up (LTFU) to a maximum of 5%. Given an alpha of 0.05, a beta of 0.80, assuming an incidence rate in the control group of 5%, a rho of 0.007 (to account for some clustering in South Sudan and Mali, based on previous relapse studies [30, 31]), and a maximum 5% LTFU, 614 post-SAM and 306 control children will be enrolled in each country, which is sufficient to detect a RR of 2.3 in South Sudan and Mali, and a RR of 2.0 in Somalia (no clustering given that all data is coming from one clinic.), and a RR of 1.7 in a combined country analysis. Given the calculations, the total number of participants enrolled in the study will be 2,760. The sample size was calculated with the use of Stata Version 13.0 software (StataCorp LP, College Station, Texas, USA).

Regarding the WASH sub-study analysis, the sub-study is considered exploratory as there is a lack of published work concerning the influence of WASH conditions, drinking water and food contamination, and enteric pathogen detection on SAM relapse rates upon which to base our estimates for calculations. Using the same conditions as for the main sample size calculation described above—a power of 0.8, an alpha of 0.05, and assuming the true proportion relapsing within 6 months of discharge to be 12%, the minimum detectable differences (MDD) between exposed and unexposed groups (e.g., proportion with/without adequate household WASH, proportion with/without enteric pathogens detected, and proportion with/without contaminated food and drinking water) under different exposure scenarios (i.e. proportion exposed from 10% – 80%) was calculated. Calculations were performed using the R package “powerMediation”, function “SSizeLogisticBin”. A sample size of 614 in each country would be adequate to detect statistically significant differences in the proportion relapsing of approximately at least 20 percentage points between groups and under different exposure scenarios. This is considered to be sufficient for an exploratory study that aims to assess poorly studied risk relationships.

After several months of study commencement, enrollment rates in Mali were significantly lower than originally anticipated. Measures were put in place to increase enrollment rates, including implementing mass screenings to identify acutely malnourished children and increasing the number of clinics involved in the study. However, these measures were insufficient to account for the low enrollment rates. It was determined that the original sample size would likely not be met within the study timeframe. Therefore, sample sizes were adjusted whereby the expected feasible sample size in Mali was reduced. To maintain sufficient power for the pooled analysis for both the main study and WASH sub-study, sample size was increased in Somalia where enrollment rates were observed to be the quickest. Also, the ratio of post-SAM children to control children in Mali was changed from 2:1 to 1:1 to increase the power for the individual country analysis given the lower expected enrollment. After these ad hoc adjustments, the new sample sizes are as follows: 800 post-SAM and 400 control children in Somalia, 614 post-SAM and 306 control children in South Sudan, and a minimum of 400 post-SAM and 400 control children in Mali, which is sufficient to detect RRs of 1.9, 2.3, and 2.2 for Somalia, South Sudan, and Mali, respectively, and an RR of 1.7 in a combined country analysis.

### Data analysis

Anthropometric indices will be computed using the WHO’s 2006 Child Growth Standards [32]. In binary analyses examining differences in participant enrollment characteristics between the post-SAM and control groups, measurements will be compared using Chi-square tests for categorical parameters and paired sample t-test and Wilcoxon sign rank test for matched continuous parameters. The analytical approach will include analyses on an individual country-level as well as using a combined dataset from all three country contexts.

Relapse rates (including relapse to SAM, MAM, and AM) will be calculated as 1) a cumulative incidence, defined as the total number of children who experience at least one episode of relapse divided by the total number of children at risk; 2) an incidence rate, defined as the total number of episodes divided by the total person-time (expressed in 100 person months); and 3) point prevalence of SAM, MAM, and AM at the different follow-up points across the six-month follow-up period. Similar indicators will be calculated for time receiving treatment, anthropometric changes, morbidity, and mortality. The cumulative probability of experiencing a relapse episode as well as the time to first episode of relapse will be compared across post-SAM and control groups using Kaplan–Meier curves and log-rank tests. See primary, secondary, and tertiary outcomes defined in Table 2. For any children LTFU, available outcome and covariates will be compared to children not lost to follow-up to determine type of missingness found in the sample.

Cox proportional hazards models will be applied to identify exposures associated with post-discharge relapse. Covariates to be included in the full regression model will be based on identified risk factors for poor outcomes after recovery from acute malnutrition in previous studies. Potential covariates will include age, sex, anthropometrics upon OTP admission and discharge, symptoms of illness during the 2 weeks before OTP admission, immunization status, infant child feeding practices, household food security, household wealth index, maternal education, whether the mother is alive, specific household WASH conditions and practices, exposure to specific pathogens via water and/or food, and presence of specific enteric pathogens at the time of SAM recovery. Crude and adjusted models will be run for all covariates to identify associated hazard ratios. The treatment center/health facility will be included as a random effect. An additional model will be run for each covariate to adjust for possible seasonal effects using 2 and 4 pie cosine and sine terms (harmonic regression). Final covariates to include in the multivariate model will be based on a combination of automated procedures (with selections based on a *p*-value < 0.2) and the existing literature.

In the pooled analysis, a random-effects model will be run where the random effect is the country, allowing the model to incorporate the different variances across studies. Each country location is weighted by the inverse of the variance of the relative risk, so that no one study drives the overall 'pooled impact'. The crude effect will be assessed, meaning the relationship between the child-level outcomes and whether the child was in the post-SAM or control group, and the adjusted effect where child- and household-level characteristics are controlled for as additional covariates. Similar analyses will be conducted on the secondary and tertiary outcomes. Also, the relationship between child linear (length/height) and ponderal (weight) growth (in both directions) will be specifically examined throughout the follow-up period and compared across the two groups.

Frequency tables and descriptive statistics will be used to describe WASH conditions, the prevalence of food and water contamination, child immune function, and the prevalence and diversity of childhood enteric infections (i.e., the prevalence of individual enteric pathogens, total number of pathogens detected, proportion children testing positive for at least one pathogen) among children discharged from CMAM treatment. Analytical statistics will be used to compare prevalence data between country locations (when applicable) and assess the association between household WASH conditions, food and water contamination, immune function, enteric infections, and the outcome of interest: the risk of relapse following discharge. All *p*-values will be two-tailed and statistical significance will be set at *p*-value < 0.05 with 95% confidence intervals. All statistical analyses for the WASH sub-study will be conducted in Stata Version 16.0 software (StataCorp, College Station, Texas, USA).

## Discussion

This study is the first of its kind to systematically track children after recovery from SAM in CMAM programs using uniform methods across multiple countries. These results will provide evidence regarding how effective and cost-efficient current implementation of the CMAM approach is in achieving sustained recovery. The study design allows the use of results to: 1) facilitate a better understanding of the burden of relapse; 2) elucidate financial costs associated with relapse; and 3) identify risk factors for relapse. Specifically, it is expected to provide a comprehensive and rigorous assessment how household WASH conditions and practices, food quality, gastrointestinal infections, using advance molecular methods to test for a wide range of enteric pathogens, influence risk of relapse across multiple settings. Furthermore, study results may help contribute to policy discussions related to developing a standard definition of relapse and common indicators for reporting and interpreting relapse rates. Findings from the WASH sub-study may help to guide how WASH interventions could be used to compliment CMAM programs to address associations between risk of infection and relapse.

With study operations occurring across multiple countries, challenges in data collection are expected and will require flexible responses to novel and ongoing context specific events. Anticipated study limitations include potentially introducing bias through community screening activities to recruit more children who may otherwise not reach CMAM programs. Additionally, we will refer children who are identified as relapsed to MAM for treatment if functioning programs are available. We anticipate the availability of MAM treatment may not be harmonized across all country locations. If we see this to be the case in many of our study locations, we will focus the discussion of findings on relapse to AM, rather than relapse specifically to SAM. We do believe these results will still be relevant to global policy around relapse given the increased attention on considering a simplified approach to addressing both SAM and MAM in on AM treatment system.

Efforts will be made to communicate the central findings and implications with study participants and communities and government officials in all three study sites. The results will be submitted for publication in peer-reviewed journals and presented through other relevant dissemination platforms. The results of this study will be submitted for publication in peer-reviewed journals and presented through other relevant dissemination platforms. The data collected in the study will be publicly available, with personal identifiable data redacted.

## Supplementary Information

**Additional file 1.** Study Data Collection Tools. File includes surveys, questionnaires and forms used in data collection throughout the study.

**Additional file 2: Supplementary Table 1.** Objective 4 Exposures. Supplementary Table 1 outlines the various WASH-related exposures that will be measured during the course of the study, their definitions, the method of collection and the frequency of collection.

**Additional file 3: Supplementary Table 2.** Objective 1 Outcomes. Supplementary Table 2 outlines the various outcomes associated with the main objective of the study, how the indicators are defined, the method of collection and the frequency of collection.

**Additional file 4: Supplementary Table 3.** Objective 3 Exposures. Supplementary Table 3 outlines the various child- and household-level factors that will be captured in data collection, their definitions, the method of collection and the frequency of collection [33].

## Data Availability

The datasets generated during this study will be available from the corresponding author upon reasonable request.

## Abbreviations

AM: Acute malnutrition
ARG: Antimicrobial resistance genes
CHW: Community health worker
CMAM: Community-based management of acute malnutrition
DBS: Dried blood spot
GAM: Global acute malnutrition
HIV: Human immunodeficiency virus
IMAM: Integrated management of acute malnutrition
IPC: Integrated food security phase classification
LTFU: Loss to follow-up
MAM: Moderate acute malnutrition
MDD: Minimum detectable differences
MUAC: Mid-upper arm circumference
ODK: Open data kit
OTP: Outpatient therapeutic programs
PCR: Polymerase chain reaction
RR: Relative risk
SAM: Severe acute malnutrition
SFP: Supplementary feeding programs
TB: Tuberculosis
WASH: Water, sanitation, and hygiene
WHZ: Weight for height z-score
