# Supplementary material for: A multi-country, prospective cohort study to measure rate and risk of relapse among children recovered from severe acute malnutrition in Mali, Somalia, and South Sudan: a study protocol
Source: BMC Nutr. 2022 Aug 24;8:90. doi: 10.1186/s40795-022-00576-x (PMC9404649; doi:10.1186/s40795-022-00576-x)
Supplement: Supplementary file 1 — Additional file 1. Study Data Collection Tools. File includes surveys, questionnaires and forms used in data collection throughout the study. [file 40795_2022_576_MOESM1_ESM.docx]

SAM Relapse Study Data Collection Tools

[Annex A – Enrollment Questionnaire 2](#_Toc25589836)

[Annex B – Clinic Follow-up Visits Anthropometric Data 13](#_Toc25589837)

[Annex C – Clinic Follow-up Visits Questionnaire 14](#_Toc25589838)

[Annex D – Semi-structured Interview Guide Regarding CMAM Program 21](#_Toc25589839)

[Annex E – Home Visit WASH Questionnaire #1 25](#_Toc25589840)

[Annex F – Home Visit WASH Questionnaire #2 32](#_Toc25589841)

[Annex G – Structured Observations of Food Preparation and Feeding Practices 33](#_Toc25589842)

## Annex A – Enrollment Questionnaire

Child ID: ___________ Child Name: __________________________________________

Male / Female Date of Birth: _______ / _______ / ___________

Day Month Year

Clinic Name: ___________________________________________ Clinic ID: ___________

Date of Visit: _______ / _______ / ___________

Day Month Year

Caregiver Name: ________________________________ Caregiver Sex: Male / Female

Enumerator Name: ________________________________ Enumerator ID: _________

**Contact Information and Home Location**

Village Name: ________________________________________

Local leader name (e.g. Village chief): ______________________

Geographical area (e.g. city, county, area): ____________________________

GPS coordinates of home: (Latitude)______________ (Longitude) ______________
*(if possible)*

Directions from the study site to the child’s home:

________________________________________________________________________________________________________________________________________________________________________________________________________________________________________________________________________________________________________________

Caregiver phone number (if applicable): _______________________

**Secondary contacts:** Can you please provide the name and phone number of at least 3 relatives and/or neighbors that we can contact in the event that we are unable to reach you? These individuals should live near your home and be someone who will likely know your whereabouts.

Secondary Contact 1:

Name: ________________________________

Relationship to the primary caregiver: _______________________________

Village Name: _________________________________

Phone number (if applicable): ____________________________

Secondary Contact 2:

Name: ________________________________

Relationship to the primary caregiver: _______________________________

Village Name: _________________________________

Phone number (if applicable): ____________________________

Secondary Contact 3:

Name: ________________________________

Relationship to the primary caregiver: _______________________________

Village Name: _________________________________

Phone number (if applicable): ____________________________

Secondary Contact 4:

Name: ________________________________

Relationship to the primary caregiver: _______________________________

Village Name: _________________________________

Phone number (if applicable): ____________________________

**Enrollment Questionnaire** Child ID: ___________

**Demographics**

I would like to ask a few questions about members of your household.

1. What is the relationship of the primary caregiver to the child?
   1. Mother *(go to #3)*
   2. Father
   3. Sibling
   4. Grandmother
   5. Other: ________________________________________
2. Is the child’s mother alive?
   1. Yes
   2. No
   3. Don’t know
3. How many years of education did the mother complete?
   1. _________ years
   2. Prefer not to answer
   3. Don’t know
4. Is the child’s father alive?
   1. Yes
   2. No
   3. Don’t know
5. Is the child a twin?
   1. Yes
   2. No
6. How many siblings does the child have? ___________
7. Where does this child fall in birth order?
   1. 1^st^ born (oldest)
   2. 2^nd^ born
   3. 3^rd^ born
   4. 4^th^ born
   5. 5^th^ born
   6. 6^th^ born
   7. 7^th^ born
   8. More than 7^th^ born. (Specify): _______

**Health and Nutrition Information**

Next, I would like to ask a few questions about the health and nutrition status for the child and other members of your household.

1. Has the child ever received any vaccinations to prevent (him/her) from getting diseases, including vaccinations received in a campaign or Child Health Day? (If possible, verify with infant’s health card.)
   1. None
   2. BCG - If yes, when? _____ / ______ / _______ DD/MM/YYYY
   3. Hepatitis B - If yes, when? _____ / ______ / _______ DD/MM/YYYY
   4. Oral Polio Vaccine - If yes, when?

_____ / ______ / _______ DD/MM/YYYY

_____ / ______ / _______ DD/MM/YYYY

_____ / ______ / _______ DD/MM/YYYY

- 1. DTaP (diphtheria, tetanus, and pertussis) vaccine - If yes, when?

_____ / ______ / _______ DD/MM/YYYY

_____ / ______ / _______ DD/MM/YYYY

_____ / ______ / _______ DD/MM/YYYY

- 1. Pneumococcal (conjugate) vaccine - If yes, when?

_____ / ______ / _______ DD/MM/YYYY

_____ / ______ / _______ DD/MM/YYYY

_____ / ______ / _______ DD/MM/YYYY

- 1. Oral Rotavirus Vaccine - If yes, when?

_____ / ______ / _______ DD/MM/YYYY

_____ / ______ / _______ DD/MM/YYYY

_____ / ______ / _______ DD/MM/YYYY

- 1. Measles / Rubella - If yes, when?

_____ / ______ / _______ DD/MM/YYYY

_____ / ______ / _______ DD/MM/YYYY

_____ / ______ / _______ DD/MM/YYYY

- 1. Others?

_______________________, When? _____ / ______ / _______ DD/MM/YYYY

_______________________, When? _____ / ______ / _______ DD/MM/YYYY

1. Has the mother been tested for HIV?
   1. Yes
   2. No (go to #12)
   3. Don’t know (go to #12)
2. What are the results?
   1. Positive
   2. Negative (go to #12)
   3. Don’t know (go to #12)
3. Is she receiving treatment?
   1. Yes
   2. No
   3. Don’t know
4. Has the child been tested for HIV?
   1. Yes
   2. No (go to #16)
   3. Don’t know (go to #16)
5. What are the results?
   1. Positive
   2. Negative (go to #16)
   3. Don’t know (go to #16)
6. Is the child receiving antibiotics?
   1. Yes
   2. No (go to #16)
   3. Don’t know (go to #16)
7. What is the name of the antibiotics? ____________________
8. Has the child ever been breastfed?
   1. Yes
   2. No (go to #19)
   3. Don’t know (go to #19)
9. Is the child currently breastfed?
   1. Yes (go to #19)
   2. No
   3. Don’t know (go to #19)
10. At what age did the child stop breastfeeding?
    1. ________ years ________months
    2. Don’t know
11. How old was the child when she/he first at food?
    1. ________ years ________months
    2. Don’t know
12. How old was the child when she/he first drank liquid other than breast milk?
    1. ________ years ________months
    2. Don’t know

1. When did the child last have diarrhea? Diarrhea means three or more loose stools per day at any point. (*Show Bristol Stool Chart*)
   1. Within the last 7 days
   2. Not within the last 7 days, but within the last month
   3. Not within the last month
   4. Don’t know
2. When did the child last suffer from fever?
   1. Within the last 7 days
   2. Not within the last 7 days, but within the last month
   3. Not within the last month
   4. Don’t know
3. When did the child last suffer from cough?
   1. Within the last 7 days
   2. Not within the last 7 days, but within the last month
   3. Not within the last month
   4. Don’t know

**Food Security**

Next, I would like to ask a few questions about experiences around food in your household.

1. In the past 4 weeks, was there ever no food to eat of any kind in your house because of lack of resources to get food?
2. Yes
3. No (go to #26)
4. Don’t know (go to #26)
5. How often did this happen?
6. Rarely (once or twice in the past four weeks)
7. Sometimes (three to ten times in the past four weeks)
8. Often (more than ten times in the past four weeks)

1. In the past 4 weeks, did you or any household member go to sleep at night hungry because there was not enough food?
2. Yes
3. No (go to #28)
4. Don’t know (go to #28)
5. How often did this happen?
6. Rarely (once or twice in the past four weeks)
7. Sometimes (three to ten times in the past four weeks)
8. Often (more than ten times in the past four weeks)
9. In the past 4 weeks, did you or any household member go a whole day and night without eating anything because there was not enough food?
10. Yes
11. No (go to #30)
12. Don’t know (go to #30)
13. How often did this happen?
14. Rarely (once or twice in the past four weeks)
15. Sometimes (three to ten times in the past four weeks)
16. Often (more than ten times in the past four weeks)

**Assistance Programs**

Next, I would like to ask about any assistance that your household might be receiving.

1. Is anyone in the child’s home currently enrolled in an assistance program that provides any of the following?
   1. Food
      1. Yes
      2. No
      3. Don’t know
   2. Cash or Vouchers
      1. Yes
      2. No
      3. Don’t know
   3. Supplies related to water, hygiene, or sanitation (e.g. water treatment tablets, jerrican, soap, any support to construct latrine)
      1. Yes
      2. No
      3. Don’t know
   4. Agricultural support (e.g. seeds, tools, etc.)
      1. Yes
      2. No
      3. Don’t know
   5. Any other assistance?
      1. Yes 🡪 (Specify) _________________________________
      2. No
      3. Don’t know

**Water and Sanitation**

Next, I would like to ask about water and toilet facilities for your household.

1. From where do you usually collect water for drinking?
   1. Surface water (river, dam, lake, pond, stream, canal, irrigation channel)
   2. Piped water into dwelling
   3. Piped water to yard/plot
   4. Piped water to neighbor
   5. Public tap/standpipe
   6. Tube well/borehole
   7. Protected dug well
   8. Unprotected dug well
   9. Protected spring
   10. Unprotected spring
   11. Rainwater
   12. Tanker-truck
   13. Car with small tank
   14. Water kiosk
   15. Bottled water
   16. Sachet water
   17. Other (Specify) _______________________________
2. Where is the water source?
   1. Inside dwelling
   2. Inside yard/ plot
   3. Elsewhere
3. Do you sometimes collect water for drinking from other sources than your usual source?
   1. Yes
   2. No
   3. Don’t know
4. What are these other sources that you use for drinking? (mark all that apply)
5. Surface water (river, dam, lake, pond, stream, canal, irrigation channel)
6. Piped water into dwelling
7. Piped water to yard/plot
8. Piped water to neighbor
9. Public tap/standpipe
10. Tube well/borehole
11. Protected well
12. Unprotected well
13. Protected spring
14. Unprotected spring
15. Rainwater
16. Tanker-truck
17. Car with small tank
18. Water kiosk
19. Bottled water
20. Sachet water
21. Other (Specify) _______________________________
22. In the last 4 weeks, how frequently has there not been sufficient drinking water for you or members of your household?
    1. Never
    2. Once or twice
    3. 2 -10 times
    4. More than 10 times
    5. Don’t know
23. What was the reason that you were unable to access water in sufficient quantities when needed?
    1. Water not available from source
    2. Source not accessible
    3. Water too expensive
    4. Don’t know
    5. Other (Specify) _______________________________
24. How long does it usually take you to go to collect water and come back home (in minutes)?
    1. ________hours ________ minutes
    2. Members do not collect
    3. Don’t know
25. Do you usually do anything to the drinking water to make it safer to drink?
26. Yes
27. No (go to #39)
28. Don’t know (go to #39)
29. What is usually done to make the water safer to drink? *(Mark all that apply.)*
30. Settling
31. Cloth filtration
32. Filtration (sand filter, ceramic filter)
33. Chlorine tablets
34. Liquid chlorine
35. Solar disinfection
36. Boiling
37. Other: ____________
38. What kind of toilet facility do members of your household usually use?
    1. Flush to piped sewer system
    2. Flush to septic tank
    3. Flush to pit latrine
    4. Flush to open drain
    5. Flush to don’t know where
    6. Ventilated improved pit latrine
    7. Pit latrine with slab
    8. Pit latrine without slab
    9. Open pit
    10. Composting toilet
    11. Bucket
    12. Hanging toilet/ latrine
    13. No facility, bush, field
    14. Other (Specify): _____________________________
39. Where is the toilet facility?
    1. Inside dwelling
    2. Inside yard/ plot
    3. Elsewhere
40. How many households share the latrine?
    1. _____________________________
    2. Don’t know

1. Where does your child usually defecate?
   1. On the ground (inside the house)
   2. In the open (outside the house)
   3. Household toilet/ latrine
   4. Nappy
   5. Potty
   6. Other
   7. Don’t know

**Socioeconomic Status**

Next, I would like to ask about things in your house.

1. Does anyone in the child’s household own any of the following in working condition?

|  |  | Yes | No | Don’t Know |
| --- | --- | --- | --- | --- |
| a. | Mattress |  |  |  |
| b. | Cell phone |  |  |  |
| c. | Refrigerator |  |  |  |
| d. | Television |  |  |  |
| e. | Radio |  |  |  |
| f. | Table |  |  |  |
| g. | Chair or bench |  |  |  |
| h. | Torch/Lantern |  |  |  |
| i. | Bicycle |  |  |  |
| j. | Car or motorcycle |  |  |  |
| k. | Mule-pulled cart |  |  |  |
| l | Shoes |  |  |  |

1. How many people live in the child’s household?

___________ (number of people)

1. How many rooms does the house have where the child lives? A room is defined as a space separated from other spaces by a wall.

__________ (number of rooms)

1. Does the house have a separate room that is used as a kitchen? A separate room is defined as having a wall that separates the kitchen from the remaining areas of the house.
   1. Yes
   2. No
   3. Don’t know
2. Does any member of the child’s household have a bank account or is part of a village savings and loans association (VSLA), savings groups, etc.?
3. Yes
4. No
5. Don’t know
6. Does your household have electricity?
7. Yes
8. No (go to #49)
9. Don’t know (go to #49)
10. What is your source of electricity?
11. Network
12. Generator
13. Solar panels
14. Batteries
15. Other: ________
16. Do you or someone living in the child’s household own this dwelling?
    1. Yes
    2. No
    3. Don’t know
17. Does any member of the child’s household own any land that can be used for agriculture?
    1. Yes
    2. No
    3. Don’t Know
18. How many hectares of agricultural land do members of the child’s household own?
    1. ______________ hectares
    2. Don’t know
19. Does any member of the child’s household own livestock?
20. Yes
21. No (End interview)
22. Don’t know (End interview)
23. Can you tell me the number of animals of each type that is owned and their use by members of the child’s household?

|  | Number (quantity) | Domestic Food Source  (eggs, meat, milk) | Income/sale of produce  (eggs, meat, milk) | Pet/companionship | Other  (specify) |
| --- | --- | --- | --- | --- | --- |
| Chicken (1) |  |  |  |  | ______ |
| Duck (2) |  |  |  |  | ______ |
| Pig (3) |  |  |  |  | ______ |
| Cattle (4) |  |  |  |  | ______ |
| Goat (5) |  |  |  |  | ______ |
| Sheep (6) |  |  |  |  | ______ |
| Horse, donkey, or mule (7) |  |  |  |  | ______ |
| Dog (8) |  |  |  |  | ______ |
| Cat (9) |  |  |  |  | ______ |
| Other (10) ___ **SPECIFY** |  |  |  |  | ______ |

1. In the home where you and the child live, what type(s) of floors are there? *(Multiple answers possible.)*
   1. Earth/sand
   2. Dung
   3. Wood planks
   4. Palm/Bamboo
   5. Parquet or polished wood
   6. Vinyl or asphalt strips
   7. Ceramic tiles
   8. Cement
   9. Carpet
   10. Other (Specify): _____________________________
2. In the home where you and the child live, what type(s) of walls are there? *(Multiple answers possible.)*
3. Cane, palm, trunks
4. Dirt
5. Bamboo with mud
6. Stone with mud
7. Uncovered adobe
8. Plywood
9. Cardboard
10. Reused wood
11. Cement
12. Stone with lime/cement
13. Bricks
14. Cement blocks
15. Covered adobe
16. Wood plants/shingles
17. Other (specify): _________________________
18. In the home where you and the child live, what type(s) of roof are there? *(Multiple answers possible.)*
19. No roof
20. Thatch, palm, leaf
21. Sod
22. Rustic mat
23. Palm, bamboo
24. Wood planks
25. Carboard
26. Metal, tin
27. Wood
28. Calamine, cement fibre
29. Ceramic tiles
30. Cement
31. Roofing shingles
32. Other (specify): ___________________________

**END INTERVIEW**

We would like to thank you for your time completing this survey. If you have any questions, you can contact us through the details listed in the informed consent forms and participant information sheet.

Do you have any questions for us now?

## Annex B – Clinic Follow-up Visits Anthropometric Data

**Clinic Name: _______________________**

Child ID: __________ Child Name: ____________________________________ Male / Female Date of Birth: ________ / ________ / _________

Day Month Year

| **Date** | **Follow-up Visit** | **MUAC** | **Weight** | **Length/ Height** | **WHZ** | **Edema** | **Outcome** | | | | | **Notes** |
| --- | --- | --- | --- | --- | --- | --- | --- | --- | --- | --- | --- | --- |
| (DD/MM/YY) | # of mos | (mm) | (kg) | (cm) |  | (0, +, ++) | Not Mal | MAM | SAM | Default | Died |  |
|  | Enrollment |  |  |  |  |  | Not Mal | MAM | SAM | Default | Died |  |
|  |  |  |  |  |  |  | Not Mal | MAM | SAM | Default | Died |  |
|  |  |  |  |  |  |  | Not Mal | MAM | SAM | Default | Died |  |
|  |  |  |  |  |  |  | Not Mal | MAM | SAM | Default | Died |  |
|  |  |  |  |  |  |  | Not Mal | MAM | SAM | Default | Died |  |
|  |  |  |  |  |  |  | Not Mal | MAM | SAM | Default | Died |  |
|  |  |  |  |  |  |  | Not Mal | MAM | SAM | Default | Died |  |
|  |  |  |  |  |  |  | Not Mal | MAM | SAM | Default | Died |  |
|  |  |  |  |  |  |  | Not Mal | MAM | SAM | Default | Died |  |
|  |  |  |  |  |  |  | Not Mal | MAM | SAM | Default | Died |  |
|  |  |  |  |  |  |  | Not Mal | MAM | SAM | Default | Died |  |
|  |  |  |  |  |  |  | Not Mal | MAM | SAM | Default | Died |  |
|  |  |  |  |  |  |  | Not Mal | MAM | SAM | Default | Died |  |

**Missed Visits:**

Scheduled Visit #: ______: 1) ______ / ______ / ______ Message? Y / N 2) ______ / ______ / ______ Message? Y / N 3) ______ / ______ / ______ Message? Y / N ☐ Default?

Scheduled Visit #: ______: 1) ______ / ______ / ______ Message? Y / N 2) ______ / ______ / ______ Message? Y / N 3) ______ / ______ / ______ Message? Y / N ☐ Default?

Scheduled Visit #: ______: 1) ______ / ______ / ______ Message? Y / N 2) ______ / ______ / ______ Message? Y / N 3) ______ / ______ / ______ Message? Y / N ☐ Default?

## Annex C – Clinic Follow-up Visits Questionnaire

Study ID: __________ Child Name: _____________________________________

Male / Female Date of Birth: _______ / _______ / ___________

Day Month Year

Clinic Name: _______________________________________ Clinic ID: ___________

Date of Follow-up Visit: _______ / _______ / ___________

Day Month Year

Caregiver Sex: __________________________________________

Enumerator Name: ________________________________ Enumerator ID: _________

**Confirm Contact Information and Home Location**  *Confirm with the caregiver the following information to see if any changes need to be made from the enrollment form.* *If not changes need to be made, then leave the following blank. If changes need to be made, complete the following.*

Village Name: ________________________________________

Local leader name (e.g. Village chief): ______________________

Geographical area (e.g. city, county, area): ____________________________

GPS coordinates of home: (Latitude)______________ (Longitude) ______________ *(if possible)*

Directions from the study site to the child’s home:

________________________________________________________________________________________________________________________________________________________________________________________________________________________________________________________________________________________

Caregiver phone number (if applicable): _______________________

**Secondary contacts:** *Confirm* *with the caregiver that the secondary contacts from the enrollment form as still applicable. If not, update by asking the following:* Can you please provide the name and phone number of 1-3 relatives and/or neighbors that we can contact in the event that we are unable to reach you? These individuals should live near your home and be someone who will likely know your whereabouts.

Secondary Contact 1:

Name: ________________________________

Relationship to the primary caregiver: _______________________________

Village Name: _________________________________

Phone number (if applicable): ____________________________

Secondary Contact 2:

Name: ________________________________

Relationship to the primary caregiver: _______________________________

Village Name: _________________________________

Phone number (if applicable): ____________________________

Secondary Contact 3:

Name: ________________________________

Relationship to the primary caregiver: _______________________________

Village Name: _________________________________

Phone number (if applicable): ____________________________

Secondary Contact 4:

Name: ________________________________

Relationship to the primary caregiver: _______________________________

Village Name: _________________________________

Phone number (if applicable): ____________________________

**Clinic Follow-up Visit Questionnaire** Child ID: ___________

**ILLNESS and HEALTHSEEKING BEHAVIORS**

I would like to ask a few questions about the child’s symptoms of illness

1. When did the child last have diarrhea? Diarrhea means three or more loose stools per day at any point. (*Show Bristol Stool Chart*)
   1. Within the last 7 days
   2. Not within the last 7 days, but within the last month
   3. Not within the last month
   4. Don’t know
2. When did the child last suffer from fever?
   1. Within the last 7 days
   2. Not within the last 7 days, but within the last month
   3. Not within the last month
   4. Don’t know
3. When did the child last suffer from cough?
   1. Within the last 7 days
   2. Not within the last 7 days
   3. Don’t know
4. When did you last seek advice or treatment for any symptoms of illness that the child had?
   1. Within the last 7 days
   2. Not within the last 7 days, but within the last month
   3. Not within the last month
   4. Don’t know

1. When did the child last receive medication for any symptoms of illness that the child had?
   1. Within the last 7 days
   2. Not within the last 7 days, but within the last month
   3. Not within the last month
   4. Don’t know
2. Where did you seek advice or treatment?

Public medical sector

- 1. Government hospital
  2. Government health center
  3. Government health post
  4. Community health worker
  5. Mobile / Outreach clinic

Private medical sector

- 1. Private hospital / clinic
  2. Private physician
  3. Private pharmacy
  4. Community Health Worker
  5. Mobile clinic
  6. Don’t know if it was Public or Private

Other source

- 1. Relative / Friend
  2. Shop / Market / street
  3. Traditional practitioner
  4. Self-treatment
  5. Other (Specify): __________________________

1. Was there a time in the last month that you wanted to take your child to the health center because of any symptom of illness, but you didn’t?
   1. Yes
   2. No (go to #9)
   3. Don’t know (go to #9)
2. If yes, why? *(Mark all that apply*)
   1. Lack of time
   2. Lack of money to pay for transport
   3. No one to watch other children
   4. Other caregivers/spouse didn’t agree on need as it is a joint decision
   5. Health center does not have capacity to treat
   6. Other (specify): _______________________________

**FOOD SECURITY**

Next, I would like to ask a few questions about experiences around food in your household.

1. In the past 4 weeks, was there ever no food to eat of any kind in your house because of lack of resources to get food?
2. Yes
3. No (go to #11)
4. Don’t know (go to #11)
5. How often did this happen?
6. Rarely (once or twice in the past four weeks)
7. Sometimes (three to ten times in the past four weeks)
8. Often (more than ten times in the past four weeks)

1. In the past 4 weeks, did you or any household member go to sleep at night hungry because there was not enough food?
2. Yes
3. No (go to #13)
4. Don’t know (go to #13)
5. How often did this happen?
6. Rarely (once or twice in the past four weeks)
7. Sometimes (three to ten times in the past four weeks)
8. Often (more than ten times in the past four weeks)
9. In the past 4 weeks, did you or any household member go a whole day and night without eating anything because there was not enough food?
10. Yes
11. No (go to #15)
12. Don’t know (go to #15)
13. How often did this happen?
14. Rarely (once or twice in the past four weeks)
15. Sometimes (three to ten times in the past four weeks)
16. Often (more than ten times in the past four weeks)

**ASSISTANCE PROGRAMS**

Next, I would like to ask about any assistance that your household might be receiving.

1. Is anyone in the child’s home currently enrolled in an assistance program that provides any of the following?
   1. Food
      1. Yes
      2. No
      3. Don’t know
   2. Cash or Vouchers
      1. Yes
      2. No
      3. Don’t know
   3. Supplies related to water, hygiene, or sanitation (e.g. water treatment tablets, jerrican, soap, support to construct latrine)
      1. Yes
      2. No
      3. Don’t know
   4. Agricultural support (e.g. seeds, tools, etc.)
      1. Yes
      2. No
      3. Don’t know
   5. Any other assistance?
      1. Yes 🡪 Specify: __________________________________
      2. No
      3. Don’t know

**END INTERVIEW**

We would like to thank you for your time completing this survey. If you have any questions, you can contact us through the details listed in the informed consent forms and participant information sheet.

Do you have any questions for us now?

## Annex D – Semi-structured Interview Guide Regarding CMAM Program

Semi-structured interview

CMAM Programmatic Factors

Nutrition Program Manager/Nutrition Head of Department

Interviewer Name: ___________________________________

Respondent Name: ___________________________________

Respondent Title: ___________________________________

Study Location: ____________________________________

Date: DD/MM/YYYY

**CMAM Protocol**

1. What are the admission criteria being implemented across the CMAM sites involved in this research study?
2. What are the discharge criteria being implemented across the CMAM sites involved in this research study?
3. What are the discharge procedures being implemented across the CMAM sites involved in this research study (e.g. minimum length of stay in the program, discharge ration of RUTF provided)?
4. Describe how closely program implementation aligns with the written CMAM protocol?
   1. What aspects align well?
   2. What aspects deviate?
   3. Can you describe why you think these deviations occur?
   4. How frequently do these deviations occur?
5. What is the frequency of visits during treatment for the CMAM sites involved in this research study (e.g. weekly, bi-weekly)?
6. Does the location of CMAM services change throughout the course of treatment (e.g. participation in OTP is in a different location than participation in the SFP)? Is this uniform across all sites involved in the research study?
7. What is the type of treatment food provided?
8. Does the type of food change throughout treatment? If so, describe the timing for the provision of each type of food (e.g. RUTF during SAM treatment in OTP and RUSF during MAM treatment in SFP)?
9. What is the dosing regimen for each of the food types provided?
10. Are any routine medical treatments provided during treatment (e.g. amoxicillin, malaria prophylaxis, measles vaccine, albendazole or mebendazole)? If so, please explain the following for each medical treatment:

- the name;
- schedule for administration;
- dose (if applicable); and
- duration of each of the medical treatments.

1. Are any other routine items provided to caregivers or patients during treatment (e.g. bednet, water treatment supplies, soap, behavior change and communication materials)? If so, please explain the following for each:

- what is provided;
- the number or amount; and
- the schedule for administration.

1. What, if any, referrals for other healthcare services occur? Please describe in detail.
2. Is any nutrition counseling routinely provided to caregivers during treatment? If so, please describe at least the following details and other relevant factors related to the counseling:

- main content and messaging;
- who conducts the counseling;
- type of counseling (group or individual); and
- timing and frequency of counseling provided

1. Describe key successes of the nutrition counseling.
2. Describe key challenges, bottlenecks, or shortcomings of the nutrition counseling.

**Non-nutrition Programs and Services**

1. Are there any other non-nutrition services or goods provided? If so, please describe.
2. Are there any parallel programs associated with the CMAM program (e.g. care groups, WASH programs)? If so, please describe.
3. Are there any food assistance programs implemented in the geographic areas in which the CMAM clinic sites involved in the research study serve? If so, please describe.
4. Are there any blanket supplementary feeding programs implemented in the geographic areas in which the CMAM clinic sites involved in the research study serve? If so, please describe.

**Referrals and Case Finding**

1. What are the different sources of referrals for admitted patients into the CMAM program? Estimate the percent of admissions that come from each of the referral sources.
2. Please describe any active case-finding through community health workers (CHWs) that occurs in the community.
3. What is the ratio of CHW to HH in the geographic areas in which the CMAM clinic sites involved in the research study serve?
4. Describe key successes of case finding and referral systems.
5. Describe key challenges, bottlenecks, or shortcomings of case finding and referral systems.

**Supply Stocks and Shortages**

1. Over the past year, what was the typical frequency and duration of stock shortages that occurred for each of the treatment food provided?
2. Was the past year a typical year in regard to the frequency and duration of stock shortages that occurred for each of the food types? If no, please explain why.
3. Over the past year, what was the typical frequency and duration of stock shortages that occurred for each of the routine medical treatments?
4. Was the past year a typical year in regard to the frequency and duration of stock shortages that occurred for each of the medical treatments? If no, please explain why.
5. Describe key successes of supplies and stock management.
6. Describe key challenges, bottlenecks, or shortcomings of supplies and stock management.

**Community Perceptions**

1. Please describe the overall community perception of the CMAM program.

1. Describe key successes of interaction and engagement of the program with the community.
2. Describe key challenges, bottlenecks, or shortcomings of interaction and engagement of the program with the community.

**Overall/General**

1. Please describe any other aspects of the actual program implementation and identify potential bottlenecks that we have not discussed that may impact program results.

## Annex E – Home Visit WASH Questionnaire #1

**Questionnaire #1 (initial home visit)**

Enumerator ID: ______________

Child sex:

Child age/DOB:

Caregiver sex:

Child ID: ______________

GPS coordinates: (Latitude)______________ (Longitude) ______________

Date of survey: DD/MM/YYYY

Time at start of survey: _____________

1. Are you the person responsible for taking care of children in this household?
   1. Yes *(go to #4)*
   2. No
2. May I speak with the person responsible for taking care of children in this household?
3. Yes *(go to #4 and continue with the children’s caregiver)*
4. No
5. When do you expect the person responsible for taking care of children in this household to come back?

_____________ *(schedule a new visit, up to twice for each household)*

1. Is your child enrolled in the SAM Relapse study?
2. Yes
3. No *(interrupt)*
4. Don’t know *(interrupt)*

**WATER ACCESS**

Next, I would like to ask you a few questions about water in your household.

*Ask the caregiver to show you where the drinking water is kept.*

1. What is the source that you used for this water for drinking?
2. Surface water (river, dam, lake, pond, stream, canal, irrigation channel)
3. Piped water into dwelling
4. Piped water to yard/plot
5. Piped water to neighbor
6. Public tap/standpipe
7. Tube well/borehole
8. Protected well
9. Unprotected well
10. Protected spring
11. Unprotected spring
12. Rainwater
13. Tanker-truck
14. Car with small tank
15. Water kiosk
16. Bottled water
17. Sachet water
18. Other (Specify) _______________________________
19. Where is that water source located?
20. In own dwelling
21. In own yard/plot
22. Elsewhere
23. Do you sometimes collect water for drinking from other sources than your usual source?
24. Yes
25. No
26. Don’t know
27. What are these other sources that you use for drinking? (mark all that apply)
28. Surface water (river, dam, lake, pond, stream, canal, irrigation channel)
29. Piped water into dwelling
30. Piped water to yard/plot
31. Piped water to neighbor
32. Public tap/standpipe
33. Tube well/borehole
34. Protected well
35. Unprotected well
36. Protected spring
37. Unprotected spring
38. Rainwater
39. Tanker-truck
40. Car with small tank
41. Water kiosk
42. Bottled water
43. Sachet water
44. Other (Specify) _______________________________
45. In the last 4 weeks, how frequently has there not been sufficient drinking water for you or members of your household?
46. Never
47. Once or twice
48. 2 -10 times
49. More than 10 times
50. Don’t know
51. What was the reason that you were unable to access water in sufficient quantities when needed?
52. Water not available from source
53. Source not accessible
54. Water too expensive
55. Don’t know
56. Other (Specify) _______________________________
57. How long does it usually take you to go to collect water and come back home (in minutes)?
58. -----------------------------------------(minutes)
59. Members do not collect
60. Don’t know
61. Did you do anything to make your water safer to drink?
62. Yes
63. No *(go to #24)*
64. Don’t know *(go to #24)*

1. What did you do to make this water safer to drink? *(Mark all that apply.)*
2. Settling
3. Cloth filtration
4. Filtration (sand filter, ceramic filter)
5. Chlorine tablets
6. Liquid chlorine
7. Solar disinfection
8. Boiling
9. Other: ____________

**SANITATION ACCESS AND CHILD FAECES DISPOSAL**

1. Can you please show me the toilet facility members of your household usually use? *(Observe and mark)*
2. Flush to piped sewer system
3. Flush to septic tank
4. Flush to pit latrine
5. Flush to open drain
6. Flush to don’t know where
7. Pit latrine
8. Ventilated improved pit latrine
9. Pit latrine with slab
10. Pit latrine without slab
11. Open pit
12. Composting toilet
13. Bucket
14. Hanging toilet
15. Hanging latrine
16. No facility, bush, field
17. Other (Specify): _____________________________
18. Where is the toilet facility?
19. In own dwelling
20. In own yard / plot
21. Elsewhere
22. How many households share the latrine?
23. ………………………..
24. Don’t know
25. Where does your child usually defecate?
26. On the ground (inside the house)
27. In the open (outside the house)
28. Household toilet/latrine
29. Nappy
30. Potty
31. Other: ____________
32. Don’t know

**ANIMAL CONTACT AND EXPOSURE**

1. Do members of this household have any contact with animals?
2. Yes, *(Below, mark all that apply)*
3. No (go to #11)
4. Don’t know (go to #11)

|  |  | Inside the house | Inside the compound | Outside the compound | None |
| --- | --- | --- | --- | --- | --- |
| a. | Chicken |  |  |  |  |
| b. | Duck |  |  |  |  |
| c. | Pig |  |  |  |  |
| d. | Cattle |  |  |  |  |
| e. | Goat |  |  |  |  |
| f. | Sheep |  |  |  |  |
| g. | Horse, donkey, or mule |  |  |  |  |
| h. | Dog |  |  |  |  |
| i. | Cat |  |  |  |  |
| J | Other (Specify)_________ |  |  |  |  |

1. Which household members have most contact with animals?
2. Yes, *(Below, mark all that apply)*
3. No (go to #11)
4. Don’t know (go to #11)

|  |  | Adult males | Adult females | Children | Other specify |
| --- | --- | --- | --- | --- | --- |
| a. | Chicken |  |  |  |  |
| b. | Duck |  |  |  |  |
| c. | Pig |  |  |  |  |
| d. | Cattle |  |  |  |  |
| e. | Goat |  |  |  |  |
| f. | Sheep |  |  |  |  |
| g. | Horse, donkey, or mule |  |  |  |  |
| h. | Dog |  |  |  |  |
| i. | Cat |  |  |  |  |
| J | Other (Specify)_________ |  |  |  |  |

1. Where do the animals sleep?

|  | Inside the house | Inside the compound | Outside the compound | Other  (specify) |
| --- | --- | --- | --- | --- |
| Chicken (1) |  |  |  | ______ |
| Duck (2) |  |  |  | ______ |
| Pig (3) |  |  |  | ______ |
| Cattle (4) |  |  |  | ______ |
| Goat (5) |  |  |  | ______ |
| Sheep (6) |  |  |  | ______ |
| Horse, donkey, or mule (7) |  |  |  | ______ |
| Dog (8) |  |  |  | ______ |
| Cat (9) |  |  |  | ______ |
| Other (10) ___ **SPECIFY** |  |  |  | ______ |

**HYGIENE AND HANDWASHING PRACTICES**

1. Can you show me where members of your household most often wash their hands?
2. Fixed facility observed (sink / tap) in dwelling
3. Fixed facility observed (sink / tap) in yard /plot
4. Mobile object observed (bucket / jug / kettle)
5. No handwashing place in dwelling / yard / plot
6. No permission to see
7. Observe if there is water available at the handwashing facility or area?
8. Yes, available
9. No, not available
10. Observe if there is soap or ash available at the handwashing facility or area?
11. Bar soap or liquid soap
12. Detergent (powder, liquid, paste)
13. Ash, mud, sand
14. No, not available
15. Do you have soap, detergent or ash available in your household to wash hands (reported availability)?
16. Yes, available
17. No, not available
18. What were all of the times when you and your household members washed your hands? (*Do not prompt. After each response, ask “any other times?”*)
19. After defecation
20. After cleaning/wiping the child’s bottom
21. After changing the nappy
22. Before preparing food
23. Before eating
24. Before feeding the child
25. After eating
26. Coming back from work/outside the home
27. Other: _______________________________
28. Cannot remember/does not know
29. Can you please show me what you used to wash your hands? *(Observe and mark all that apply.)*
30. Water only
31. Soap and water
32. Sand and water
33. Ash and water
34. Other: _____________

**WATER TREATMENT AND STORAGE**

1. We would like to collect a sample of the water you give to your child to test for the presence of bacteria. Can you please provide me with a cup of water that your child drinks from?
2. Yes *(proceed with water sample collection)*
3. No / refuse *(End Interview)*
4. How much water do you estimate this household uses each day (for all activities)?
5. How much water (in litres) is stored in your house for drinking?
6. Can you please show me where you keep your water for drinking? (Observe and select the container type where the drinking water is stored by the household including volumes and covers, and mark which water container the sample came from).

| Container type | Mark which container the water sample originates from with an “X” | Covered | Uncovered | Approximate volume of container (in litres) |
| --- | --- | --- | --- | --- |
| Clay pot |  |  |  |  |
| Jerrycan |  |  |  |  |
| Plastic bucket |  |  |  |  |
| Large barrel |  |  |  |  |
| Buried clay reservoir |  |  |  |  |
| Plastic bottles |  |  |  |  |
| Unable to observe |  |  |  |  |
| Other…………. |  |  |  |  |

*[Note to interviewer: Collect water direct from the receptacle from the tap or with a cup the household uses. Ask the interviewee to help you. Please ensure the sample bag is labelled with DATE, TIME and CHILD ID NUMBER]*

1. Measure turbidity (NTU): _______
2. Measure free chlorine residual (mg/L): ______

**FOOD PREPARATION**

Next, I would like to ask you about food prepared for and consumed by the child.

1. What liquids did the child enrolled in the study consume yesterday, during the day and night?
2. Breastmilk
3. Plain water
4. Animal milk (cow, goat, camel)
5. Tea
6. Juice
7. Broth
8. Formula
9. Others: __________
10. What types of food did the child consume yesterday during the day and night?
11. *For each kind, ask:* Was it cooked or raw?

| **Type of food** | **What kind?** | **Preparation** |
| --- | --- | --- |
| Staples |  | Cooked Raw |
| Dairy |  | Cooked Raw |
| Fruit |  | Cooked Raw |
| Vegetable |  | Cooked Raw |
| Meat |  | Cooked Raw |
| Fish |  | Cooked Raw |
| Other: ______ |  | Cooked Raw |

1. What do you usually do to fresh fruit or vegetables before giving them to your child? *(Select all that apply.)*
   1. Wash
   2. Disinfect/bleach
   3. Peel
   4. Cook
   5. Other: __________
   6. Don’t know
   7. Nothing
2. What time(s) throughout the day do you usually prepare food for the child? *(Mark all that apply)*
3. Early morning before sunrise
4. First thing after sunrise
5. Mid-morning
6. Mid-day
7. Mid-afternoon
8. Early evening before sunset
9. Late evening after sunset
10. During the night
11. How often do you re-serve food later in the day that was prepared earlier in the day?
12. Never (go to #17)
13. Sometimes (once per week)
14. Daily
15. Don’t know (go to #17)
16. For how long is prepared food usually stored before all of it is used?
17. Do not store prepared food
18. Less than 1 day
19. 1-2 days
20. More than 2 days
21. Don’t know
22. Can you please show me where you keep the food that you give to your child? *Observe: What type of container is it? Does it contain raw food? Is the container covered/open/sealed? (Select all that apply.)*

| **Type of container** | **Raw food?** | **The container is…** |
| --- | --- | --- |
| Plastic container | Yes No | Covered Open Sealed |
| Metallic container | Yes No | Covered Open Sealed |
| Clay pot | Yes No | Covered Open Sealed |
| Bag | Yes No | Covered Open Sealed |
| Fridge | Yes No | Covered Open Sealed |
| Other: ________ | Yes No | Covered Open Sealed |

1. Do you do anything to prepare food before serving it again? *(Ask “anything else?” and check all that apply.)*
2. Nothing
3. Reheat
4. Boil
5. Mix with water/milk/other liquid
6. Mix with freshly prepared food
7. Other: _________

*If you have been instructed to collect a food sample, proceed with the following section. If you have not been instructed to collect a food sample, go to* ***END OF THE SURVEY****.*

1. Do you have prepared food in the house today?
2. Yes
3. No (go to #21)
4. We would like to collect a sample of the food to test for the presence of bacteria. May I have a sample (e.g. 2 spoonfuls) of food as you would give to your child?
5. Yes *(proceed with food collection)*
6. No / refuse / no food available

*[Note to interviewer: Collect up to 3 food types with a spoon borrowed from the household. Ask the interviewee to help you. Please ensure the food sample bag is labelled with DATE, TIME and CHILD ID NUMBER]*

1. Record the types of food collected:
   1. Food type 1: ____________________________
   2. Food type 2: ____________________________
   3. Food type 3: ____________________________

**END INTERVIEW**

We would like to thank you for your time completing this survey. If you have any questions, you can contact us through the details listed in the informed consent forms and participant information sheet.

Do you have any questions for us now?

## Annex E – Home Visit WASH Questionnaire #2

**Questionnaire # 2 (second and third home visits)**

Enumerator ID: ______________

Child sex:

Child age/DOB:

Caregiver sex:

Child ID: ______________

GPS coordinates: (Latitude)______________ (Longitude) ______________

Date of survey: DD/MM/YYYY

Time at start of survey: _____________

1. Are you the person responsible for taking care of children in this household?
2. Yes *(go to #4)*
3. No
4. May I speak with the person responsible for taking care of children in this household?
5. Yes *(go to #4 and continue with the children’s caregiver)*
6. No
7. When do you expect the person responsible for taking care of children in this household to come back?

_____________ *(schedule a new visit, up to twice for each household)*

1. Is your child enrolled in the SAM Relapse study?
2. Yes
3. No *(interrupt)*
4. Don’t know *(interrupt)*

**WATER ACCESS**

Next, I would like to ask you a few questions about water in your household.

*Ask the caregiver to show you where the drinking water is kept.*

1. What is the source that you used for this water for drinking?
2. Surface water (river, dam, lake, pond, stream, canal, irrigation channel)
3. Piped water into dwelling
4. Piped water to yard/plot
5. Piped water to neighbor
6. Public tap/standpipe
7. Tube well/borehole
8. Protected well
9. Unprotected well
10. Protected spring
11. Unprotected spring
12. Rainwater
13. Tanker-truck
14. Car with small tank
15. Water kiosk
16. Bottled water
17. Sachet water
18. Other (Specify) _______________________________
19. Where is that water source located?
20. In own dwelling
21. In own yard/plot
22. Elsewhere
23. Do you sometimes collect water for drinking from other sources than your usual source?
24. Yes
25. No
26. Don’t know
27. What are these other sources that you use for drinking? (mark all that apply)
28. Surface water (river, dam, lake, pond, stream, canal, irrigation channel)
29. Piped water into dwelling
30. Piped water to yard/plot
31. Piped water to neighbor
32. Public tap/standpipe
33. Tube well/borehole
34. Protected well
35. Unprotected well
36. Protected spring
37. Unprotected spring
38. Rainwater
39. Tanker-truck
40. Car with small tank
41. Water kiosk
42. Bottled water
43. Sachet water
44. Other (Specify) _______________________________
45. In the last 4 weeks, how frequently has there not been sufficient drinking water for you or members of your household?
46. Never
47. Once or twice
48. 2 -10 times
49. More than 10 times
50. Don’t know
51. What was the reason that you were unable to access water in sufficient quantities when needed?
52. Water not available from source
53. Source not accessible
54. Water too expensive
55. Don’t know
56. Other (Specify) _______________________________
57. How long does it usually take you to go to collect water and come back home (in minutes)?
58. -----------------------------------------(minutes)
59. Members do not collect
60. Don’t know
61. Did you do anything to make your water safer to drink?
62. Yes
63. No *(go to #24)*
64. Don’t know *(go to #24)*

1. What did you do to make this water safer to drink? *(Mark all that apply.)*
2. Settling
3. Cloth filtration
4. Filtration (sand filter, ceramic filter)
5. Chlorine tablets
6. Liquid chlorine
7. Solar disinfection
8. Boiling
9. Other: ____________

**ANIMAL CONTACT AND EXPOSURE**

1. Do members of this household have any contact with animals?
2. Yes, *(Below, mark all that apply)*
3. No (go to #11)
4. Don’t know (go to #11)

|  |  | Inside the house | Inside the compound | Outside the compound | None |
| --- | --- | --- | --- | --- | --- |
| a. | Chicken |  |  |  |  |
| b. | Duck |  |  |  |  |
| c. | Pig |  |  |  |  |
| d. | Cattle |  |  |  |  |
| e. | Goat |  |  |  |  |
| f. | Sheep |  |  |  |  |
| g. | Horse, donkey, or mule |  |  |  |  |
| h. | Dog |  |  |  |  |
| i. | Cat |  |  |  |  |
| J | Other (Specify)_________ |  |  |  |  |

1. Which household members have most contact with animals?
2. Yes, *(Below, mark all that apply)*
3. No (go to #11)
4. Don’t know (go to #11)

|  |  | Adult males | Adult females | Children | Other specify |
| --- | --- | --- | --- | --- | --- |
| a. | Chicken |  |  |  |  |
| b. | Duck |  |  |  |  |
| c. | Pig |  |  |  |  |
| d. | Cattle |  |  |  |  |
| e. | Goat |  |  |  |  |
| f. | Sheep |  |  |  |  |
| g. | Horse, donkey, or mule |  |  |  |  |
| h. | Dog |  |  |  |  |
| i. | Cat |  |  |  |  |
| J | Other (Specify)_________ |  |  |  |  |

1. Where do the animals sleep?

|  | Inside the house | Inside the compound | Outside the compound | Other  (specify) |
| --- | --- | --- | --- | --- |
| Chicken (1) |  |  |  | ______ |
| Duck (2) |  |  |  | ______ |
| Pig (3) |  |  |  | ______ |
| Cattle (4) |  |  |  | ______ |
| Goat (5) |  |  |  | ______ |
| Sheep (6) |  |  |  | ______ |
| Horse, donkey, or mule (7) |  |  |  | ______ |
| Dog (8) |  |  |  | ______ |
| Cat (9) |  |  |  | ______ |
| Other (10) ___ **SPECIFY** |  |  |  | ______ |

**HYGIENE AND HANDWASHING PRACTICES**

1. Can you show me where members of your household most often wash their hands?
2. Fixed facility observed (sink / tap) in dwelling
3. Fixed facility observed (sink / tap) in yard /plot
4. Mobile object observed (bucket / jug / kettle)
5. No handwashing place in dwelling / yard / plot
6. No permission to see
7. Observe if there is water available at the handwashing facility or area?
8. Yes, available
9. No, not available
10. Observe if there is soap or ash available at the handwashing facility or area?
11. Bar soap or liquid soap
12. Detergent (powder, liquid, paste)
13. Ash, mud, sand
14. No, not available
15. Do you have soap, detergent or ash available in your household to wash hands (reported availability)?
16. Yes, available
17. No, not available
18. What were all of the times when you and your household members washed your hands? (*Do not prompt. After each response, ask “any other times?”*)
19. After defecation
20. After cleaning/wiping the child’s bottom
21. After changing the nappy
22. Before preparing food
23. Before eating
24. Before feeding the child
25. After eating
26. Coming back from work/outside the home
27. Other: _______________________________
28. Cannot remember/does not know
29. Can you please show me what you used to wash your hands? *(Observe and mark all that apply.)*
30. Water only
31. Soap and water
32. Sand and water
33. Ash and water
34. Other: _____________

**WATER TREATMENT AND STORAGE**

1. We would like to collect a sample of the water you give to your child to test for the presence of bacteria. Can you please provide me with a cup of water that your child drinks from?
2. Yes *(proceed with water sample collection)*
3. No / refuse *(End Interview)*
4. How much water do you estimate this household uses each day (for all activities)?
5. How much water (in litres) is stored in your house for drinking?
6. Can you please show me where you keep your water for drinking?
7. (Observe and select the container type where the drinking water is stored by the household including volumes and covers, and mark which water container the sample came from).

| Container type | Mark which container the water sample originates from with an “X” | Covered | Uncovered | Approximate volume of container (in litres) |
| --- | --- | --- | --- | --- |
| Clay pot |  |  |  |  |
| Jerrycan |  |  |  |  |
| Plastic bucket |  |  |  |  |
| Large barrel |  |  |  |  |
| Buried clay reservoir |  |  |  |  |
| Plastic bottles |  |  |  |  |
| Unable to observe |  |  |  |  |
| Other…………. |  |  |  |  |

*[Note to interviewer: Collect water direct from the receptacle from the tap or with a cup the household uses. Ask the interviewee to help you. Please ensure the sample bag is labelled with DATE, TIME and CHILD ID NUMBER]*

1. Measure turbidity (NTU): _______
2. Measure free chlorine residual (mg/L): ______

**FOOD PREPARATION**

Next, I would like to ask you about food prepared for and consumed by the child.

1. What liquids did the child enrolled in the study consume yesterday, during the day and night?
2. Breastmilk
3. Plain water
4. Animal milk (cow, goat, camel)
5. Tea
6. Juice
7. Broth
8. Formula
9. Others: __________
10. What types of food did the child consume yesterday during the day and night?
11. *For each kind, ask:* Was it cooked or raw?

| **Type of food** | **What kind?** | **Preparation** |
| --- | --- | --- |
| Staples |  | Cooked Raw |
| Dairy |  | Cooked Raw |
| Fruit |  | Cooked Raw |
| Vegetable |  | Cooked Raw |
| Meat |  | Cooked Raw |
| Fish |  | Cooked Raw |
| Other: ______ |  | Cooked Raw |

1. What do you usually do to fresh fruit or vegetables before giving them to your child? *(Select all that apply.)*
2. Wash
3. Disinfect/bleach
4. Peel
5. Cook
6. Other: __________
7. Don’t know
8. Nothing
9. What time(s) throughout the day do you usually prepare food for the child? *(Mark all that apply)*
10. Early morning before sunrise
11. First thing after sunrise
12. Mid-morning
13. Mid-day
14. Mid-afternoon
15. Early evening before sunset
16. Late evening after sunset
17. During the night
18. How often do you re-serve food later in the day that was prepared earlier in the day?
19. Never (go to #17)
20. Sometimes (once per week)
21. Daily
22. Don’t know (go to #17)
23. For how long is prepared food usually stored before all of it is used?
24. Do not store prepared food
25. Less than 1 day
26. 1-2 days
27. More than 2 days
28. Don’t know
29. Can you please show me where you keep the food that you give to your child? *Observe: What type of container is it? Does it contain raw food? Is the container covered/open/sealed? (Select all that apply.)*

| **Type of container** | **Raw food?** | **The container is…** |
| --- | --- | --- |
| Plastic container | Yes No | Covered Open Sealed |
| Metallic container | Yes No | Covered Open Sealed |
| Clay pot | Yes No | Covered Open Sealed |
| Bag | Yes No | Covered Open Sealed |
| Fridge | Yes No | Covered Open Sealed |
| Other: ________ | Yes No | Covered Open Sealed |

1. Do you do anything to prepare food before serving it again? *(Ask “anything else?” and check all that apply.)*
2. Nothing
3. Reheat
4. Boil
5. Mix with water/milk/other liquid
6. Mix with freshly prepared food
7. Other: _________

*If you have been instructed to collect a food sample, proceed with the following section. If you have not been instructed to collect a food sample, go to* ***END OF THE SURVEY****.*

1. Do you have prepared food in the house today?
2. Yes
3. No (go to #21)
4. We would like to collect a sample of the food to test for the presence of bacteria. May I have a sample (e.g. 2 spoonfuls) of food as you would give to your child?
5. Yes *(proceed with food collection)*
6. No / refuse / no food available

*[Note to interviewer: Collect up to 3 food types with a spoon borrowed from the household. Ask the interviewee to help you. Please ensure the food sample bag is labelled with DATE, TIME and CHILD ID NUMBER]*

1. Record the types of food collected:
   1. Food type 1: ____________________________
   2. Food type 2: ____________________________
   3. Food type 3: ____________________________

**END INTERVIEW**

We would like to thank you for your time completing this survey. If you have any questions, you can contact us through the details listed in the informed consent forms and participant information sheet.

Do you have any questions for us now?

## Annex G – Structured Observations of Food Preparation and Feeding Practices

Enumerator ID: ______________

Child ID: _______________

Date of survey: DD/MM/YYYY ________________

Time observation begins: HH:MM __________

Time observations ends: HH:MM __________

*Notes for enumerator:*

1. *Position yourself in an unobtrusive location where you have a clear view of the food preparation and serving activities (move as necessary).*
2. *Record information about the preparation, cooking, serving and storage of child’s food*
3. *At the end of the observations, record the availability of a handwashing station, and availability of soap, ash and water at the handwashing station*
4. *Notes on how to be an observer:*
   1. *Introduce yourself politely to the household and any attending people*
   2. *Be polite and friendly with the participants but keep the conversation and interaction to a minimum. You are here to observe but not to participate.*
   3. *Do not show your form to participants or other people before the end of the observation period (and then do it only if asked by the study participants)*
   4. *Write down everything you are not sure about and discuss it with your supervisor later.*
   5. *There is no need to invent, if nothing happens, nothing happens!*

**Food observations**

1. Who is involved in making the child’s food?
   1. Only the identified caregiver
   2. The caregiver and another person/s (specify who this is): _________________
   3. Another person/s, not the caregiver (specify who this is): _________________
2. Is previously cooked food being given to the child?
   1. Yes (go to #10)
   2. No
3. What food is being prepared (specify) for the child?

_________________________________________________

1. What are the main ingredients used and how were they stored (ask caregiver for any information needed)? (Check all that apply)

| **Ingredient  (specify each)** | **Quantity** | **Origin** | **Storage location**  a. Refrigerator  (or on ice)  b. Freezer  c. Inside house, room temperature  d. Outside, room temperature | **Storage**  a. Covered  b. Not covered  c. Not applicable |
| --- | --- | --- | --- | --- |
|  |  |  | 🞏 a 🞏b 🞏c 🞏d | 🞏 a 🞏b 🞏c |
|  |  |  | 🞏 a 🞏b 🞏c 🞏d | 🞏 a 🞏b 🞏c |
|  |  |  | 🞏 a 🞏b 🞏c 🞏d | 🞏 a 🞏b 🞏c |
|  |  |  | 🞏 a 🞏b 🞏c 🞏d | 🞏 a 🞏b 🞏c |
|  |  |  | 🞏 a 🞏b 🞏c 🞏d | 🞏 a 🞏b 🞏c |
|  |  |  | 🞏 a 🞏b 🞏c 🞏d | 🞏 a 🞏b 🞏c |
|  |  |  | 🞏 a 🞏b 🞏c 🞏d | 🞏 a 🞏b 🞏c |

**During Preparation**

1. Are hands washed before preparing food?
   1. Yes, with soap and water
   2. Rinsed with only water
   3. No
2. Are ingredients washed before preparing food?

| **Ingredient** | **Washed with detergent and water?** | **Cleaned with only water** | **Not cleaned** |
| --- | --- | --- | --- |
|  | 🞏 | 🞏 | 🞏 |
|  | 🞏 | 🞏 | 🞏 |
|  | 🞏 | 🞏 | 🞏 |
|  | 🞏 | 🞏 | 🞏 |
|  | 🞏 | 🞏 | 🞏 |
|  | 🞏 | 🞏 | 🞏 |

1. What utensils are used to prepare food and are they cleaned before use?

| **Utensil (list all)** | **Cleaned with detergent and water?** | **Cleaned with only water** | **Not cleaned** |
| --- | --- | --- | --- |
|  | 🞏 | 🞏 | 🞏 |
|  | 🞏 | 🞏 | 🞏 |
|  | 🞏 | 🞏 | 🞏 |
|  | 🞏 | 🞏 | 🞏 |
|  | 🞏 | 🞏 | 🞏 |
|  | 🞏 | 🞏 | 🞏 |

1. What surface is the food being prepared on?
   1. Raised surface inside house
   2. Floor inside house
   3. Raised surface outside of house
   4. Floor outside house
2. Was the surface cleaned (disinfected) before preparation?
   1. Yes, with soap or disinfectant
   2. Yes, with only water
   3. No
3. Has the food been previously prepared?
   1. Yes
   2. No (go to #12)
4. What food is the previously prepared food being served to the child (specify)?

_________________________________________________

1. What are the main ingredients used and how were they stored (ask caregiver for any information needed)? (Check all that apply)

| **Ingredient  (specify each)** | **Quantity** | **Origin** | **Storage location**  a. Refrigerator  (or on ice)  b. Freezer  c. Inside house, room temperature  d. Outside, room temperature | **Storage**  a. Covered  b. Not covered  c. Not applicable |
| --- | --- | --- | --- | --- |
|  |  |  | 🞏 a 🞏b 🞏c 🞏d | 🞏 a 🞏b 🞏c |
|  |  |  | 🞏 a 🞏b 🞏c 🞏d | 🞏 a 🞏b 🞏c |
|  |  |  | 🞏 a 🞏b 🞏c 🞏d | 🞏 a 🞏b 🞏c |
|  |  |  | 🞏 a 🞏b 🞏c 🞏d | 🞏 a 🞏b 🞏c |
|  |  |  | 🞏 a 🞏b 🞏c 🞏d | 🞏 a 🞏b 🞏c |
|  |  |  | 🞏 a 🞏b 🞏c 🞏d | 🞏 a 🞏b 🞏c |
|  |  |  | 🞏 a 🞏b 🞏c 🞏d | 🞏 a 🞏b 🞏c |

1. Where was this food stored?
   1. Refrigerator
   2. Freezer
   3. Sealed container at room temperature
   4. Loosely covered container (including covered plate or bowl) at room
       temperature
   5. Uncovered at room temperature
2. Is this food being heated?
   1. Yes
   2. No *(go to Q17)*
3. Is food heated to boiling before serving?
   1. Yes
   2. No *(Go to Q17)*
4. Are any of the following added to the food before or during cooking/reheating?
   1. Water
   2. Milk
   3. Tea
   4. Other: ___________________
   5. None of the above

**Before feeding**

1. Are any of the following added to the food after cooking/reheating?
   1. Water
   2. Milk
   3. Tea
   4. Other: ___________________
   5. None of the above
2. Are these liquids heated to boiling before they are added?
   1. Yes
   2. No
   3. N/A
3. Are caregiver’s hands washed before feeding child?
   1. Yes, with soap and water
   2. Only rinsed with water
   3. No
4. Are child’s hands washed before eating?
   1. Yes, with soap and water
   2. Only rinsed with water
   3. No

**Feeding**

1. Who is involved in child feeding?
   1. Only the identified caregiver
   2. The caregiver and another person/s (specify who this is)______________
   3. Another person/s, not the caregiver (specify who this is) _________________
2. Where is child seated?
   1. Child seated on bare floor
   2. Child is seated on mat on the floor
   3. Child is seated on caregiver’s knee
   4. Child seated in high chair
   5. Other
3. Is the surface that the child is eating on cleaned before they eat?
   1. Yes, with detergent
   2. Yes, with only water
   3. No
4. How is food served to the child?
   1. Food served to child from separate plate or bowl
   2. Food served to child from cooking vessel
   3. Food served by caregiver’s hand
   4. Food served by spoon or another utensil
   5. Child feeds themselves with their hands
5. Are the utensils being washed before eating?
   1. Yes, with soap and water
   2. Only rinsed with water
   3. No
6. Are any drinks given to child in addition to food?
   1. Water
   2. Milk
   3. Tea
   4. Other: ___________________
7. Are drinks provided in cups that have been washed before drinking?
   1. Yes, with soap and water
   2. Only rinsed with water
   3. No

**Storage**

1. Is excess food stored away?
   1. Yes
   2. No
2. Is hot food cooled before it is stored?
   1. Yes
   2. No
3. How is the food cooled?
   1. Table inside
   2. Outside
4. How is excess food stored?
   1. Excess food is discarded
   2. Excess food is stored in refrigerator or freezer
   3. Excess food is returned to cooking vessel
   4. Excess food is stored in sealed container
   5. Excess food is stored in loosely covered container, including covered plate/bowl
   6. Excess food stored uncovered at room temperature

**Contamination**

1. Are live animals near to the food preparation area?
   1. Yes
   2. No
2. Are live animals near to where the child is fed?
   1. Yes
   2. No
3. If yes to Q32 and Q33, please specify the animals:

………………………………………………………….

1. Are raw foods and cooked foods kept separately?
   1. Yes
   2. No

**Availability of handwashing facility and materials**

1. Observe where members of your household wash their hands?
   1. Fixed facility observed (sink / tap) in dwelling
   2. Fixed facility observed (sink / tap) in yard /plot
   3. Mobile object observed (bucket / jug / kettle)
   4. No handwashing place in dwelling / yard / plot
   5. No permission to see
2. Observe if there is water available at the handwashing facility or area?
   1. Yes, available
   2. No, not available
3. Observe if there is soap or ash available at the handwashing facility or area?
   1. Bar soap or liquid soap
   2. Detergent (powder, liquid, paste)
   3. Ash, mud, sand
   4. No, not available
4. Observe if the household has soap, detergent or ash available to wash hands (observed availability)?
   1. Shown
   2. Not shown

**We would like to thank you for your time completing this survey and letting us observe you and your child. If you have any questions, you can contact us through the details listed in the informed consent forms and participant information sheet.**

**Do you have any questions for us now?**

**…………………………………………**
