# Supplementary material for: A multi-country, prospective cohort study to measure rate and risk of relapse among children recovered from severe acute malnutrition in Mali, Somalia, and South Sudan: a study protocol
Source: BMC Nutr. 2022 Aug 24;8:90. doi: 10.1186/s40795-022-00576-x (PMC9404649; doi:10.1186/s40795-022-00576-x)
Supplement: Supplementary file 2 — Additional file 2: Supplementary Table 1. Objective 4 Exposures. Supplementary Table 1 outlines the various WASH-related exposures that will be measured during the course of the study, their definitions, the method of collection and the frequency of collection. [file 40795_2022_576_MOESM2_ESM.docx]

**Supplementary Table 1**. Objective 4 Exposures

| **Objective 4:** To identify WASH-related exposures associated with SAM relapse | | | | |
| --- | --- | --- | --- | --- |
| **Exposure** | **Indicator** | **Definition/description** | **Data Collection Method** | **Data Collection Frequency** |
| Household WASH conditions and practices | Water, sanitation, and hygiene practices (various indicators) and animal exposures | e.g., source of household drinking water; time to collect drinking water; treatment of drinking water; type of water container used; presence and type of container cover; frequency of water collection; type of sanitation facility/practice; disposal of child feces; critical times of handwashing; use of soap for handwashing; animal ownership, exposure to animals and contact | Enrollment and follow up survey (caregiver recall via questionnaire) and WASH HH questionnaire, | Enrollment, 3 months follow-up, and 6 months follow-up |
| Food preparation | Food preparation (various indicators) | e.g., types of food consumed by the child; whether foods were cooked or raw; type of container used to store food; whether container is covered; food used for multiple meals per day; time prepared food is stored; reheating of food before reserving food | Enrollment and follow up survey (caregiver recall via questionnaire), WASH HH questionnaire and structured observations of food preparation | Enrollment and across the follow-up period |
| Household water quality | *E. coli* and total coliforms | Colony-forming units (CFU) per 100 mL | Sample of drinking water at household | Enrollment, 3 months follow-up, and 6 months follow-up |
|  | Turbidity | Nephelometric Turbidity Units (NTU) |  |  |
|  | Free chlorine residual | mg/L |  |  |
| Household food quality | *E. coli*, total coliforms | Colony-forming units (CFU) per gram | Sample of food at household | Enrollment and across the follow-up period |
|  | Selected enteropathogens detection | Presence of any one of 30 pathogens^1^ |  |  |
| Enteric pathogen presence in rectal swabs | Selected enteropathogens detection | Presence of any one of 30 potential pathogens^1^ | Rectal swab from child at clinic | Enrollment |
| Antimicrobial resistance | Antimicrobial resistant genes (ARGs) | Presence of selected, clinically relevant ARGs based on ARGs detection via qPCR assays (to be determined) | Rectal swab from child at clinic, secondary data collection of SAM and relapse treatment data and sample of food at household | Enrollment and across the follow-up study period |
| Enteric pathogen presence in dired blood spots (DBS) | Selected enteropathogens detection | Presence any one of 30 potential pathogens^1^ | Dried blood spots at the clinic | Dried blood spots at OTP enrolment, mid-way through treatment, discharge, 1 month and 4 month post discharge |

^1^These include but are not limited to: Adenovirus 40/41, rotavirus A, norovirus GI/GII, *Salmonella* spp. (including serovars Typhi and Paratyphi), *Campylobacter* spp. (*C. jejuni, C. coli, C. lari*), *Shigella* spp. (*S. boydii*, *S. sonnei, S. flexneri, S.dysenteriae*), *Clostridium difficile* Toxin A/B, enterotoxigenic *Escherichia coli* (ETEC) LT/ST, *E. coli* O157, Shiga-like toxin-producing *E. coli,*  stx1/stx2, *Yersinia enterocolitica*, *Vibrio cholerae*, *Giardia lamblia*, *Entamoeba histolytica*, and *Cryptosporidium* spp. (*C. parvum, C. hominis*).
