# Supplementary material for: A multi-country, prospective cohort study to measure rate and risk of relapse among children recovered from severe acute malnutrition in Mali, Somalia, and South Sudan: a study protocol
Source: BMC Nutr. 2022 Aug 24;8:90. doi: 10.1186/s40795-022-00576-x (PMC9404649; doi:10.1186/s40795-022-00576-x)
Supplement: Supplementary file 3 — Additional file 3: Supplementary Table 2. Objective 1 Outcomes. Supplementary Table 2 outlines the various outcomes associated with the main objective of the study, how the indicators are defined, the method of collection and the frequency of collection. [file 40795_2022_576_MOESM3_ESM.docx]

**Supplementary Table 2**. Objective 1 Outcomes

| **Objective 1:** to compare the cumulative incidence of SAM, MAM, and AM among children after recovery from SAM in outpatient treatment programs with the cumulative incidence of SAM among children who did not previously experience acute malnutrition | | | | |
| --- | --- | --- | --- | --- |
| **Outcome** | **Indicator** | **Definition/description** | **Data Collection Method** | **Data Collection Frequency** |
| Post-discharge relapse rates (to SAM, MAM, and AM)^1^ | Cumulative incidence (incidence proportion) of SAM, MAM, and AM^1^ | the total number of children who experience at least one episode of relapse to SAM, MAM, and AM^1^ over 6 months divided by the total number of children at risk | Anthropometric assessments | 1-, 2-, 3-, 4-, 5-, and 6-months follow-up |
|  | Incidence rate (incidence density) of SAM, MAM, and AM^1^ | the total number of SAM, MAM, and AM^1^ episodes divided by the total person-time (expressed in 100 person months) |  |  |
|  | Point prevalence of children with SAM, MAM, and AM^1^ | the total number of children with SAM, MAM, and AM^1^ at each of the follow-up points: 1-, 2-, 3-, 4-, 5-, and 6-months divided by the total number of children at each of the follow-up points: 1-, 2-, 3-, 4-, 5-, and 6-months |  |  |
| Time receiving treatment for SAM, MAM, and AM | Average number of weeks receiving treatment for SAM, MAM, and AM | The total number of weeks children are enrolled in OTP/SFP for treatment of SAM and/or MAM divided by 26 weeks (i.e., the 6-month follow-up period) divided by the total number of children | Programmatic data | Duration of the 6-month follow-up period |
| Post-discharge mortality | Cumulative incidence (incidence proportion) of mortality | the total number of children who died over 6 months divided by the total number of children at risk | Caregiver recall | 1, 2, 3-, 4-, 5-, and 6-months follow-up |
|  | Incidence rate (incidence density) of mortality | the total number of deaths divided by the total person-time (expressed in 100 person months) |  |  |
| Post-discharge morbidity^2^ | Cumulative incidence (incidence proportion) of morbidity^2^ | the total number of children who experience at least one day with symptoms of illness (diarrhea, cough, or fever) in the 7 days prior to each follow-up visit over 6 months divided by the total number of children at risk | Caregiver recall | 1-, 2-, 3-, 4-, 5-, and 6-months follow-up |
|  | Prevalence of morbidity^2^ | the total number of children who experience at least one day with a symptom of illness (diarrhea, cough, or fever) in the 7 days prior to each follow-up visits at: 1-, 2-, 3-, 4-, 5-, and 6-months divided by the total number of children at each of the follow-up visits: 1-, 2-, 3-, 4-, 5-, and 6-months | Caregiver recall | 1-, 2-, 3-, 4-, 5-, and 6-months follow-up |
| Post-discharge changes in anthropometric measurements (height/length, weight, MUAC, HAZ, WAZ, and WHZ) | Change in height/length^3^ over 6 months following SAM recovery | Height/length^3^ at 6 months follow-up subtracted by height/length upon enrollment | Height/length^3^ | Enrollment and 6 months follow-up |
|  | Growth velocity^3^ over 6 months following SAM recovery | Height/length^3^ at 6 months follow-up subtracted by height/length upon enrollment  Divide this number by 12 (i.e., total number of months of follow-up) | Height/length^3^ | Enrollment, 1-, 2-, 3-, 4-, 5-, and 6-months follow-up |
|  | Growth velocity^3^ over month preceding each follow-up visit | Height/length^3^ at each follow-up visit subtracted by the height/length at the preceding follow-up visit. Divide this number by the number of months between the two time points. | Height/length^3^ | Enrollment, 1-, 2-, 3-, 4-, 5-, and 6-months follow-up |

^1^SAM will be defined as MUAC < 115 mm, WHZ < -3, and/or edema; MAM will be defined as MUAC ≥ 115 mm and < 125 mm, WHZ ≥ -3 and < -2, with no edema; AM will be defined as MUAC < 125 mm, WHZ < - 2, and/or edema.

^2^Morbidity will be defined as experiencing at least one illness symptom (diarrhea, fever, cough) during the 7 days prior to each of the scheduled 1-, 2-, 3-, 4-, 5-, and 6-month follow-up visits.

^3^In addition to linear growth measured by height/length, the same calculations will be run for changes in growth for weight, MUAC, HAZ, WAZ, and WHZ.
