# Supplementary material for: A multi-country, prospective cohort study to measure rate and risk of relapse among children recovered from severe acute malnutrition in Mali, Somalia, and South Sudan: a study protocol
Source: BMC Nutr. 2022 Aug 24;8:90. doi: 10.1186/s40795-022-00576-x (PMC9404649; doi:10.1186/s40795-022-00576-x)
Supplement: Supplementary file 4 — Additional file 4: Supplementary Table 3. Objective 3 Exposures. Supplementary Table 3 outlines the various child- and household-level factors that will be captured in data collection, their definitions, the method of collection and the frequency of collection [33]. [file 40795_2022_576_MOESM4_ESM.docx]

**Supplementary Table 3.** Objective 3 Exposures

| **Objective 3:** to identify child- and household-level factors associated with SAM relapse | | | | |
| --- | --- | --- | --- | --- |
| **Exposure** | **Indicator(s)** | **Definition/description** | **Data Collection Method** | **Data Collection Frequency** |
| Individual child characteristics | Child age, sex, and birth order | Age (months); sex (male/female); birth order (1^st^, 2^nd^, 3^rd^, etc.). | Enrollment and follow up survey (caregiver recall via questionnaire) | Enrollment |
|  | Anthropometric measurements upon initial SAM recovery | MUAC (mm); height/length (cm); weight (kg); presence of bilateral edema | Anthropometric measurement and clinical assessment | Enrollment |
|  | Child health history (various indicators) | e.g., known HIV status of mother; known HIV status of child; members in household being treated for TB | Enrollment and follow up survey (caregiver recall via questionnaire) | Enrollment |
|  | Child’s recent symptoms of illness and medical treatment (various indicators) | e.g., in past 7 days, the number of days child experienced diarrhea; in past 7 days, the number of days child experienced fever; in past 7 days, the number of days child experienced cough; medical treatment in the past month; reason for medical treatment | Enrollment and follow up survey (caregiver recall via questionnaire) and secondary data collection of SAM and relapse treatment data | Enrollment, 1-, 2-, 3-, 4-, 5-, and 6-months follow-up |
|  | Infant and young child feeding practices | Continued breastfeeding and introduction of complementary foods | Enrollment and follow up survey (caregiver recall via questionnaire) | Enrollment |
| Household level characteristics | Household demographics (various indicators) | e.g., caregiver age; relationship of caregiver to child; child’s father is living; child’s mother is living; number of siblings to child | Anthropometric measurement and clinical assessment | Enrollment |
|  | Food Security | Household Hunger Scale [28] | Enrollment and follow up survey (caregiver recall via questionnaire) | Enrollment 1-, 2-, 3-, 4-, 5-, and 6-months follow-up |
|  | Participation in assistance programs (various indicators) | e.g., member of the household receiving food assistance; member of the household participating in cash program; | Enrollment and follow up survey (caregiver recall via questionnaire) | Enrollment 1-, 2-, 3-, 4-, 5-, and 6-months follow-up |
| Socioeconomic Status (SES) | SES Index | Based on an SES index by Psaki et al. [33] WAMI Index and is comprised of four main components: water and sanitation, assets, maternal education and HH income | Enrollment and follow up survey (caregiver recall via questionnaire) and WASH HH questionnaire | Enrollment |
